# Infrared thermographic evaluation of cleaning stone relics at tomb of Yue Fei

**DOI:** 10.1371/journal.pone.0359835

**Published:** 2026-10-08

**Authors:** Ting Zhang, Sheng Ye, Liang Ye, Zekai Qian

**Affiliations:** 1 Lian Heng Memorial Hail, Administration of Yue Fei’Temple Scenic Area, Hangzhou, China; 2 School of Civil and Architectural Engineering, Zhejiang University of Science and Technology, Hangzhou, China; National Autonomous University of Mexico Institute of Geophysics: Universidad Nacional Autonoma de Mexico Instituto de Geofisica, MEXICO

## Abstract

This study investigates the efficacy and safety of steam cleaning technology for the removal of surface contaminants from stone cultural relics at the Tomb of Yue Fei, a nationally protected heritage site in China. The research focuses on common pollutants including dust accumulation, surface encrustation, hydro erosion, bioerosion, and anthropogenic graffiti. An evaluation method based on infrared thermography is proposed to assess cleaning effectiveness by analyzing temperature variation (T) before and after treatment. Field experiments were conducted using a steam cleaner with controlled parameters (pressure, temperature, angle, distance, and duration), and cleaning outcomes were evaluated through visual inspection, chromatic aberration measurement, and microscopic imaging. Results demonstrate that steam cleaning effectively removes most contaminants without causing significant damage to the stone substrate. The study also provides a set of recommended steam cleaning parameters for different pollutant types on limestone surfaces. The findings validate the feasibility of using infrared thermography as a non-destructive evaluation tool for cleaning efficacy, contributing to the development of standardized protocols for the conservation of stone heritage.

## 1. Introduction

The tomb of Yue Fei is located at the southern foot of Qixia Mountain, West Lake, and was designated as one of the first batch of key cultural relics under national protection in 1961. Field investigations indicate that multiple stone cultural relics at the site suffer from structural deformation, moisture accumulation at the base, acid rain exposure, soluble salts, and root penetration by vegetation—factors collectively contributing to co-occurring contaminant types that threaten long-term preservation. On-site surveys identify two primary categories of surface contaminants: water-induced damage and biological deterioration. Water-induced damage accounts for approximately 50% of all observed contamination: surface runoff causes discoloration and contaminant deposition (20%); capillary water leads to accumulation of soluble salts and formation of rust-like crusts (15%); and condensate results in water staining on rock surfaces (15%). The remaining contaminants include dust accumulation, surface encrustation, and anthropogenic graffiti.The current status and spatial distribution of typical pollutants at tomb of Yue Fei are presented in Fig 1.

**Fig 1:**
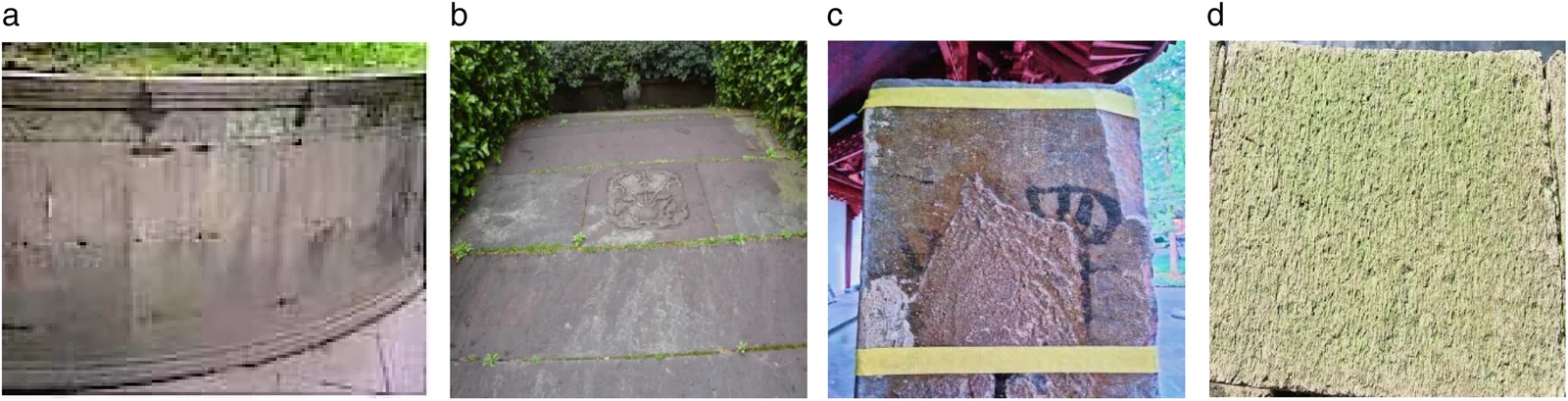
Current contaminants map of Tomb of Yue Fei. (a) Surface encrustation, (b) Hydro erosion, (c) Ink Contaminants, (d) Bioerosion.

To further characterize the composition and formation mechanisms of these pollutants,representative samples were collected from the stone surface and analyzed in the laboratory.X-ray diffraction analysis of the surface crust revealed a complex mixture of natural weathering products and exogenous sediments,with primary components including 51.2% calcium carbonate,20.4% calcium oxalate,12.8% carbonate, and 9.6% quartz.pH test strips conducted on water samples from the Yue Fei Temple area showed alkaline conditions (pH 7–8) with sulfate ion concentrations reaching 200 mg/L.Calcium compounds formed on rock surfaces due to floating dust and contaminants caused surface pollution and discoloration.Microscopic examination of biologically eroded areas identified fungal hyphae,lichen bodies,and moss roots.As summarized in Table 1, these analytical results provide a scientific basis for classifying surface pollution types.

**Table 1:** Evaluation of surface contaminants on stone cultural relics of Tomb of Yue Fei.

Types of Contaminants	Distribution area	Combination type	Main ingredients	Necessity of cleaning
Dust accumulation	All	Adsorption type	Calcium sulfate and other dust particles	Needs cleaning
Surface encrustation	Tomb of Yue Fei, Half-Moon Pond fence, Aliform wall, Jingzhong Bridge, other inscriptions	Tight coupling	Compounds such as quartz, calcium sulfate, and calcium carbonate	Needs cleaning
Hydro erosion	Tomb of Yue Fei, Jingzhong Bridge, Half-Moon Pond fence, tomb tower, other inscriptions	Intrusive type	Sulfate ions and calcium ions, etc.	Needs cleaning
Anthropogenic graffiti	Jingzhong Cypress Pavilion	Intrusive type	Carbon black particles	Needs cleaning
Bioerosion	Tomb of Yue Fei, tomb tower, Jingzhong Bridge, Half-Moon Pond fence,other inscriptions	Tight coupling	Mold, lichen, moss, etc.	Needs cleaning

This study focuses on stone cultural relics at tomb of Yue Fei, investigating the efficacy of steam cleaning technology in removing various existing pollutants. Field measurements were conducted to assess cleaning performance, and the results were evaluated using a standardized steam cleaning effectiveness evaluation method. This validation confirms the feasibility of the evaluation method and provides theoretical support for determining optimal technical parameters and safety criteria for steam cleaning of common surface contaminants on lithologies analogous to those found at tomb of Yue Fei.

In recent years, with advances in science and technology and heightened national emphasis on cultural heritage conservation, conventional cleaning methods have increasingly failed to meet current conservation objectives and principles. Steam cleaning—a green, environmentally benign, highly efficient, and operationally straightforward technique—has thus gained favor among heritage conservation practitioners [1]. He [2] reviewed the development history, operational mechanisms, and practical applications of steam cleaning technology, providing foundational information for Chinese conservation professionals. Zhang [3] demonstrated that steam jet cleaning exhibits measurable efficacy for stone carvings and historic building surfaces, with relatively low moisture penetration; however, its effectiveness against deeply embedded black crusts remains limited. Zhao [4] observed in on-site cleaning experiments that steam cleaning effectively removes loosely adhered contaminants such as ink stains and surface dust. Early research into steam cleaning technology was inspired by foreign technicians’ observation that hot steam could efficiently remove oil residues from garage floors [5]. Holden Conservation Ltd. (UK) [6] applied low-pressure steam cleaning to the London Monument and found it highly effective in removing black crusts from stone carvings, with no visible surface damage.

By establishing a theoretical analysis model for steam cleaning technology and integrating it with practical cleaning operations, the influence of technical parameters such as steam temperature, pressure, and velocity on the surface of stone cultural relics was simulated, thereby obtaining targeted reference data [7]. International scholars have conducted a series of research analyses on steam cleaning technology, finding that high-temperature steam has a sterilizing effect on microorganisms. When the steam injection time is between three to five seconds, it can eliminate over 50% of stubborn bacteria [8]. Yan et al. [9] utilized fluid simulation software to analyze the flow state of a steam nozzle, determining the optimal cleaning distance and subsequently developing a corresponding cleaning machine.

Some domestic scholars have investigated steam cleaning technology using numerical simulation methods. For example, Xu [10] employed numerical calculation methods to study the impact of hot steam injection on cascade outlet parameters. The study found that, with a fixed injection angle, the steam injection flow rate is proportional to the nozzle width; a smaller width results in a weaker flow rate. Zhao et al. [11] conducted a numerical simulation analysis of the technical parameters of steam cleaning technology. They proposed a corresponding cleaning process based on the effective action parameters for cleaning and the safe control range of steam, providing a reference for the removal of surface contaminants from sandstone cultural relics.

In summary, steam cleaning technology offers advantages such as safety, non-Contaminants, and effective cleaning when applied to the conservation of stone cultural relics, making it a promising technique for this purpose. However, in practical applications, attention must be paid to operational details and the selection of technical parameters to ensure cleaning efficacy while minimizing potential damage to the cultural relics themselves.

The cleaning of stone cultural relics should adhere to the principles of “preserving the original state” and “minimal intervention.” To ensure the cleaning process is non-destructive and effective, non-destructive testing technology must be employed to assess changes on the relic surface before and after cleaning [12]. Establishing evaluation indices for stone cultural relics requires setting up a scientific and reasonable index system, where the evaluation indices should be purposeful, scientific, and feasible [13,14].

The method of using ultrasonic testing to assess the weathering degree of stone cultural relics is currently well-established. Chen [15] utilized the high resolution and non-destructive characteristics of ultrasound tomography technique to evaluate rock weathering. Sun [16] analyzed the dispersion characteristics of ultrasonic transmission waves and Rayleigh surface waves, measured the velocity of longitudinal ultrasonic waves, and combined these with other methods to verify the reliability of ultrasonic testing results. Ultrasonic technology is also employed to assess the weathering measurement and damage degree of stone cultural relics [17]. International researchers have integrated ultrasonic methods with other detection techniques, such as moisture content measurement, to conduct safety evaluations of rock surface damage and weathering extent [18,19].

Furthermore, temperature change serves as a significant index for examining the rock surface deterioration condition. Chen [20] found that under high temperatures, the intrinsic stress of rock increases, and cyclic temperature fluctuations damage the rock mass structure, while stress concentration occurs at water-bearing fissures in rock under low temperatures. Shao [21] utilized infrared thermal imaging technology to evaluate the weathering degree of stone cultural relics. The results indicated that greater temperature amplitude correlates with more severe deterioration, thereby validating the feasibility of infrared detection for assessing rock weathering.

Methods for assessing the cleaning effectiveness of surface contaminants on stone cultural relics through color change are also relatively common. Zhou et al. [22] determined the cleaning effectiveness based on the chromatic aberration results and microscopic roughness change results before and after two cleaning cycles. Wang [23] evaluated the cleaning effect by naked eye observation and weight detection of chromatic aberration changes. The results indicated that after cleaning, the test blocks exhibited a reddish hue, a reduction in weight, and a relatively increased chromatic aberration change, suggesting a favorable cleaning outcome. Mauro Matteini et al. [24] employed ion chromatography to detect the cleaning effectiveness on stone cultural relics of a city gate. Zina-Sabrina et al. [25] adopted a novel spectral imaging method to detect the deep cleaning of stone surface contaminants, which can effectively reflect the cleaning depth and thereby achieve high-precision detection.

Zeng [26] evaluated the desalination effect through three methods: mass change, porosity detection, and ultrasonic testing, thereby determining the degree of removal efficacy for different desalination materials. Yan [27] employed multiple scientific techniques to assess the cleaning effectiveness in the cleaning of stone carving diseases. These included microscopic observation to examine the microstructural changes on the cultural relic surface after desalination, and the use of a spectrophotometer to measure the chromatic aberration in contaminated areas before and after cleaning, which served to evaluate the cleaning outcome.

Internationally, research groups from France, Italy, and Greece have made substantial contributions to the application of infrared thermography (IRT) in stone heritage conservation. Moropoulou and Avdelidis [28] pioneered the use of IRT for moisture monitoring and conservation assessment in historic structures, demonstrating its effectiveness for evaluating cleaning interventions and protective treatments on porous stone facades. The National Technical University of Athens research group established a comprehensive framework for integrating IRT with other non-destructive techniques for the diagnosis and protection of built cultural heritage [29]. In Italy, Pappalardo et al. [30] applied IRT combined with photogrammetric three-dimensional models to evaluate different weathering types affecting natural stones in heritage buildings, demonstrating that thermal contrasts correlate with surface morphology, color, and environmental conditions. Grinzato et al. [31] analyzed the feasibility of using IRT to detect sub-surface stratification and detachments in frescoes from the perspective of thermal inertia. Biscarin et al. [32] proposed a multi-sensing approach combining UAV photogrammetry, infrared thermography, and ground-penetrating radar for enhanced structural and material degradation assessment of the Roman masonry bridge of Ponte Lucano. Carlomagno et al. [33] systematically reviewed the application of infrared thermography and geophysical techniques in cultural heritage conservation, establishing theoretical foundations for thermal measurement interpretation.In France, Vázquez and colleagues [34] conducted experimental thermo-hydric studies to simulate natural weathering conditions, using IRT to monitor microstructural changes induced by thermal cycling and moisture migration in building stones. Their work highlighted the importance of emissivity calibration and environmental control in obtaining reliable thermographic data for weathering assessment. More recently, Ibarra-Castanedo et al. [35] introduced solar loading thermography for the inspection of civil engineering and cultural heritage structures, utilizing time-lapsed thermographic surveys and advanced signal processing to detect subsurface defects under natural solar excitation.

## 2. Materials and methods

### 2.1. Evaluation method of steam cleaning effect on stone cultural relics based on infrared thermography

#### 2.1.1. Establishment of evaluation method for steam cleaning effect using infrared thermography detection.

(1) Evaluation of steam cleaning impact on stone cultural relics damage using infrared images

The steam cleaning effect can be directly assessed through infrared images and three-dimensional temperature distribution maps. The results of safety tests on steam cleaning of stone cultural relics indicate that cleaning time, angle, and pressure are positively correlated with the temperature increase range, while cleaning distance is inversely correlated with the temperature increase range. The appropriate cleaning time should be controlled within 5 minutes. A good cleaning effect is associated with good rock permeability and uniform temperature distribution; minor rock damage is accompanied by small temperature changes before and after cleaning.

In addition, the surface temperature change of rocks is influenced by factors such as environmental temperature and humidity, light intensity, wind speed, rock material, and different types of contaminants. Therefore, attention should be paid to temperature changes during cleaning testing, and appropriate steam cleaning technical parameters should be determined to ensure uniform distribution of rock surface temperature.To ensure the reliability of thermographic measurements, the emissivity of the stone surface was set to 0.95 based on literature values for limestone materials.To minimize the impact of solar radiation, all measurements were conducted in overcast or shaded areas, with environmental temperature and humidity recorded. The interval between thermal imaging before and after cleaning was maintained at approximately 30 minutes to ensure comparability of environmental conditions.

(2) Evaluation method of steam cleaning effect based on temperature variation T

The test results of steam cleaning limestone test blocks show that the temperature increase range after cleaning differs between clean test block surfaces and those with contaminants. Clean test blocks exhibit a small temperature increase range after cleaning, cool down quickly, and can rapidly return to the initial temperature range; whereas test blocks with contaminants show a large temperature increase range and a small temperature decrease range after cleaning.A larger Temperature variation before and after cleaning in clean areas corresponds to a greater degree of damage to the test block, which is demonstrated in indoor cleaning tests.

Based on the temperature change amplitudes before and after cleaning under different cleaning parameters and for different contaminants, combined with the results of chromatic aberration detection and microscopic image detection, the cleaning effect of contaminants on the surface of stone cultural relics is evaluated using the initial surface temperature of the rock before cleaning as t_1_, the surface temperature after cleaning as t_2_, and the Temperature variation T before and after cleaning as the index, where positive values indicate temperature rise and negative values indicate temperature drop. The calculation formula is as follows (1):


$$\begin{array}{c} T=\frac{\left(t_{\text{2}}-t_{\text{1}}\right)}{t_{\text{1}}}\times 100\text{\%} \end{array}$$ (1)

T denotes the magnitude of temperature change before and after cleaning (%), and t_1_ and t_2_ represent the temperatures before and after cleaning (°C), respectively.

#### 2.1.2. Feasibility analysis of the evaluation method using temperature variation T.

Currently, there is no unified or fixed evaluation criteria for assessing the cleaning effectiveness of surface contaminants on stone cultural relics. Existing detection indicators for temperature are primarily used to evaluate the degree of surface weathering or the presence of cracks in the rock. Therefore, this study employs data fitting based on the detection results of chromatic aberration and roughness to analyze temperature variation T.

(1) Applicability test of using temperature variation T to evaluate cleaning effectiveness

Data fitting was performed between the temperature variation T and the calculated chromatic aberration ΔE1 as well as the surface kurtosis Sku, respectively. The fitting function models is $Y=Ae^{\frac{-x}{B}}+y_{\text{0}}$ and the fitting results are shown in Fig 2.

**Fig 2:**
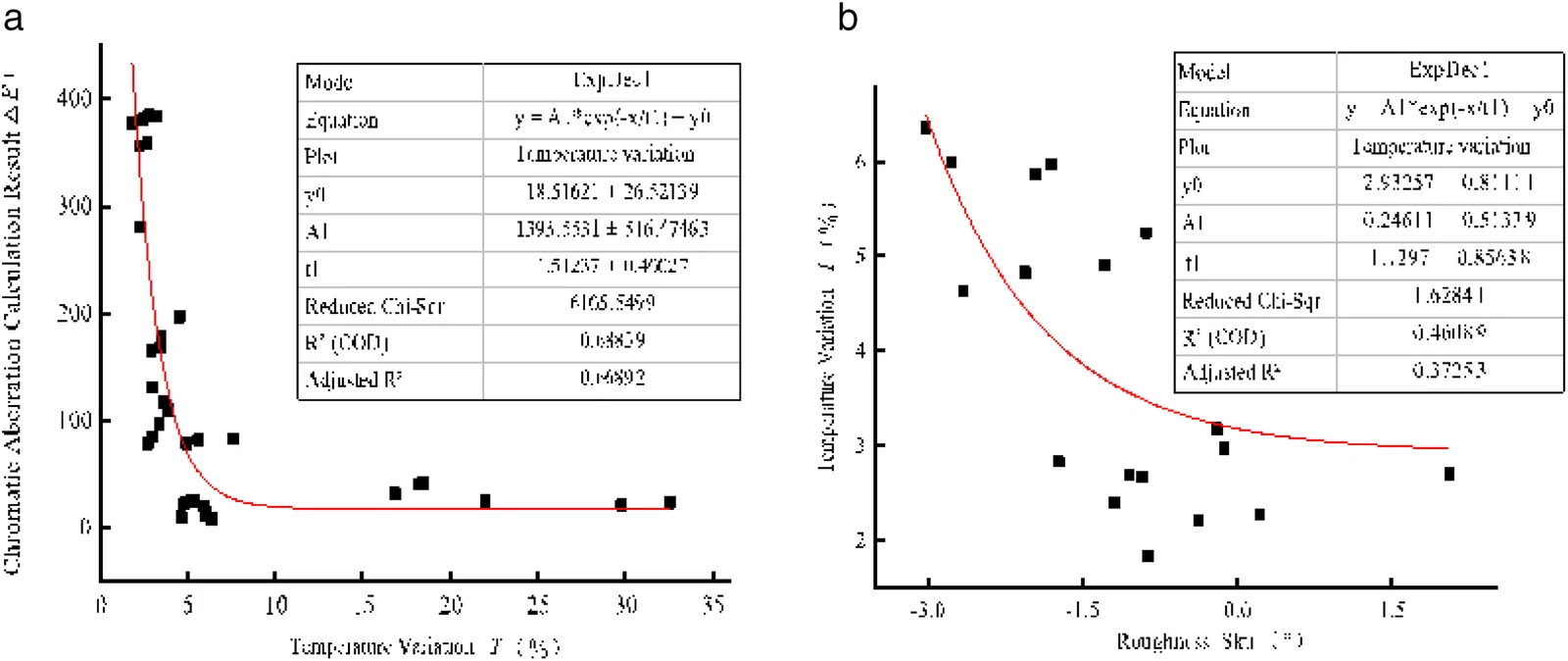
Fitting results of temperature variation T and chromatic aberration change. (a) The fitting results of temperature variation T and chromatic aberration calculation result △E1, (b) Fitting results of temperature variation T and surface roughness Sku.

The fitting formula for temperature variation T and the calculated chromatic aberration ΔE1 is:


$$\begin{array}{c} Y=7.61055{\text{e}}^{\frac{-\text{x}}{-16.03798}}+83.98673 \end{array}$$ (2)

Y represents the calculated chromatic aberration ΔE1, and x denotes the temperature variation T. The key fitting factor R^2^ is 0.69.

The fitting formula for temperature variation T and surface kurtosis Sku is:


$$\begin{array}{c} Y=0.24611{\text{e}}^{\frac{-\text{x}}{1.1297}}+2.93257 \end{array}$$ (3)

Y represents the temperature variation T, and x denotes the surface kurtosis Sku. The key fitting factor R^2^ is 0.46.

(1) Feasibility of Using Temperature Variation T to Evaluate Cleaning Effectiveness

The fitting results between temperature variation T and surface kurtosis Sku indicate that the key fitting factor R^2^ ranges from 0.36 to 0.46, suggesting a weak correlation between the two parameters. In contrast, the key fitting factor R^2^ for the relationship between temperature variation T and chromatic aberration change ranges from 0.53 to 0.69, indicating a stronger association.Therefore, the method of using the temperature variation T to evaluate cleaning effectiveness demonstrates a certain degree of feasibility. The primary source of temperature measurement error stems from differences in ambient temperature.

### 2.2. Steam cleaning test plan for surface contaminants on stone cultural relics at Tomb of Yue Fei

As tomb of Yue Fei is a semi-open tourist site, the cleaning test must be conducted during periods of low visitor traffic and within fenced-off areas to ensure unimpeded implementation of the cleaning work. The test period spans one month, from mid-May to mid-June 2024. This cleaning primarily targets surface contamination and deterioration, including dirt deposits, water damage, and biological colonization. Deionized water is selected as the cleaning agent. A comprehensive cleaning will be carried out following an analysis of the test results and safety assessment.

(1) Cleaning test equipment

The primary cleaning equipment and inspection tools are listed in Table 2.

**Table 2:** Equipment list for the cleaning test at Tomb of Yue Fei.

Classification	Instrument name	Version	Operational parameter
Cleaning equipment	Steam cleaner	KY-H10	Steam pressure: 2 ~ 8 bar, steam temperature: 120 ~ 140°C
Testing tool	Precision chromatic aberration meter (3nm)	3NH-NR110	Light source: D65, aperture: 8 mm, detection temperature: 0°C ~ 40°C
	Hygrograph	JT-C50	Detection range: 0% ~ 40%, Operating environment: −5°C ~ 60°C, Instrument accuracy: ± 0.5%, Detection depth: 50 mm
	Portable microscope	DDL-M1	Magnification: 1 × 400
	Infrared thermal imager	FOTRIC 326Q	Temperature measurement range: −20°C ~ 120°C; 0°C ~ 350°C, with a precision of ±2°C or 2%.
Cleaning materials	Deionized water (resistivity ≥1 MΩ-cm), brush, spray bottle		

The equipment listed in Table 2 was selected to comprehensively evaluate cleaning efficacy: the precision colorimeter quantified color changes (ΔE) before and after cleaning; the hygrograph monitored surface moisture content; the portable microscope provided direct visualization of contaminant removal and surface morphology; and the infrared thermal imager assessed temperature variations to detect potential substrate damage. These complementary techniques ensure a robust assessment of both cleaning effectiveness and safety.

(2) Cleaning procedure

First, the contaminated surface of the stone cultural relic is uniformly sprayed with deionized water using a spray bottle to prepare for cleaning. Next, high-temperature steam is applied for rapid cleaning, assisted by a soft-bristled brush; cleaning parameters are selected according to the type of contaminant to prevent thermal stress damage caused by non-uniform heating of the stone. Finally, the cleaned area is rinsed with clean water to remove residual contaminants, allowed to air-dry naturally, and then inspected; any incompletely cleaned areas undergo a second cleaning cycle.

## 3. Results

### 3.1. Steam cleaning results for different contaminants on the Tomb of Yue Fei

#### 3.1.1. Dust accumulation cleaning results.

Dust contaminants on stone surfaces are predominantly loosely accumulated on the surface and thus relatively easy to remove. For stone cultural relics featuring fine surface textures—such as incised or relief carvings—the texture characteristics must be considered during cleaning. In the case of incised features, auxiliary cleaning with a soft-bristled brush is required, followed by steam application at an angle aligned with the direction of the texture.The cleaning parameters selected for this operation were as follows: nozzle angle of 30°, standoff distance of 1 cm, steam pressure of 4 bar, and dwell time ranging from 1 to 3 minutes.Colorimetric analysis of the dust accumulation area revealed a significant increase in brightness after cleaning: the luminance difference ΔL increased by 209%, and the total color difference △E1 between the cleaned and uncleaned areas was 12.56.The coverage value Z, defined as the percentage of the cleaned area exhibiting chromatic values within 5% of the reference clean stone surface, was 55.3%, indicating that most of the dust had been removed, restoring the original stone color.The results are presented in Fig 3.

**Fig 3:**
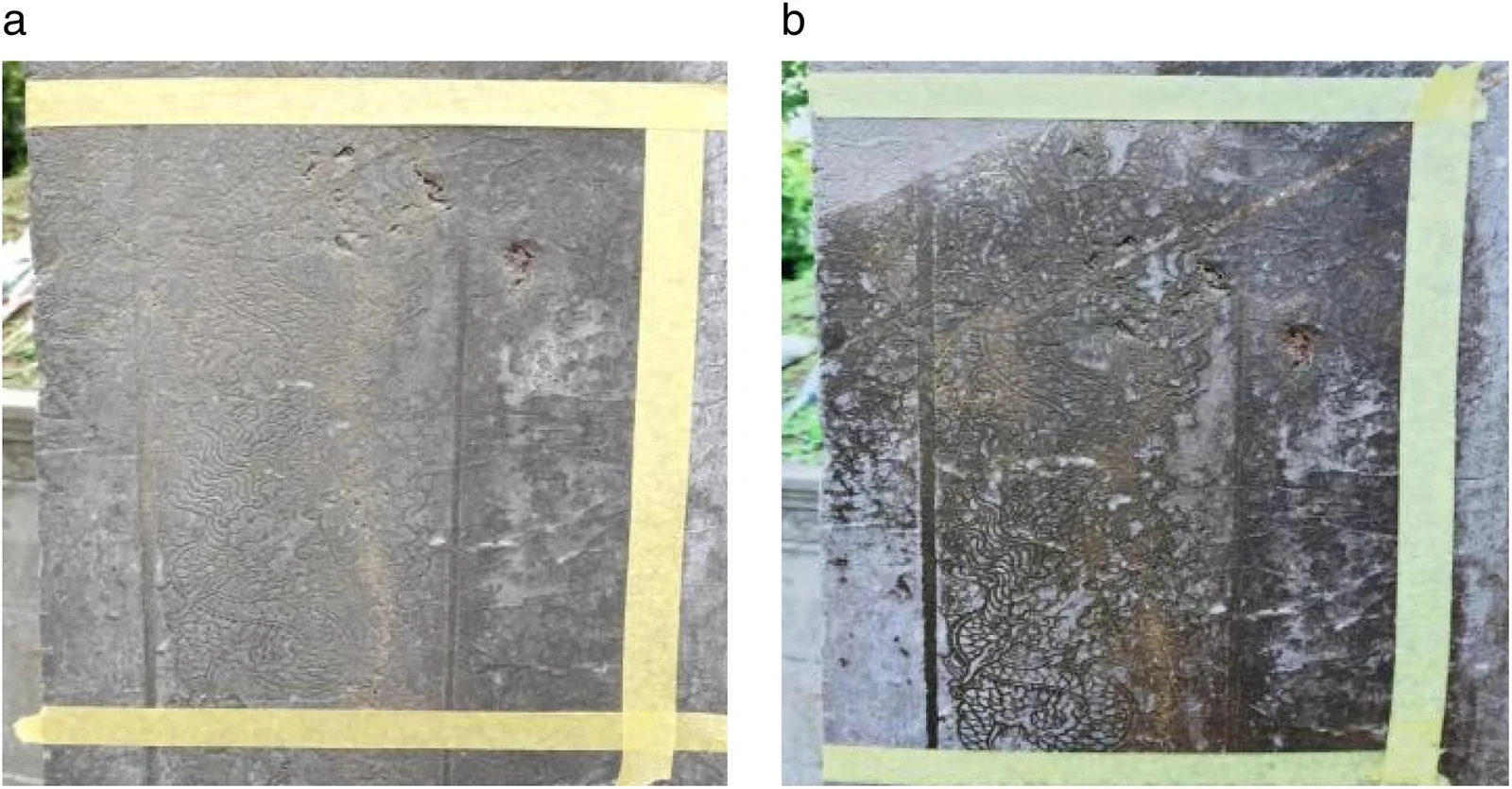
Local cleaning results of dust accumulation on the tombstone of Yue Fei. (a) Tombstone of Yue Fei-Before cleaning, (b) Tombstone of Yue Fei-After cleaning.

#### 3.1.2. Cleaning results of surface crust.

For the earthen crust on the surface of the tomb structure, which has a relatively dense contaminant structure and a thick covering layer, cleaning should first involve moistening it with hot steam. The cleaning nozzle should then be positioned perpendicular to the contaminated surface at a 90-degree angle, with a distance of 3 cm, a pressure of 4 bar, and a duration of 1 minute to achieve the removal effect.For the surface crust on Yue Fei’s tomb, the color difference between the cleaned area and a reference clean area was 12.00, indicating that the crust was effectively removed with only minor color deviation from the original stone surface.The cleaning results are shown in Fig 4.

**Fig 4:**
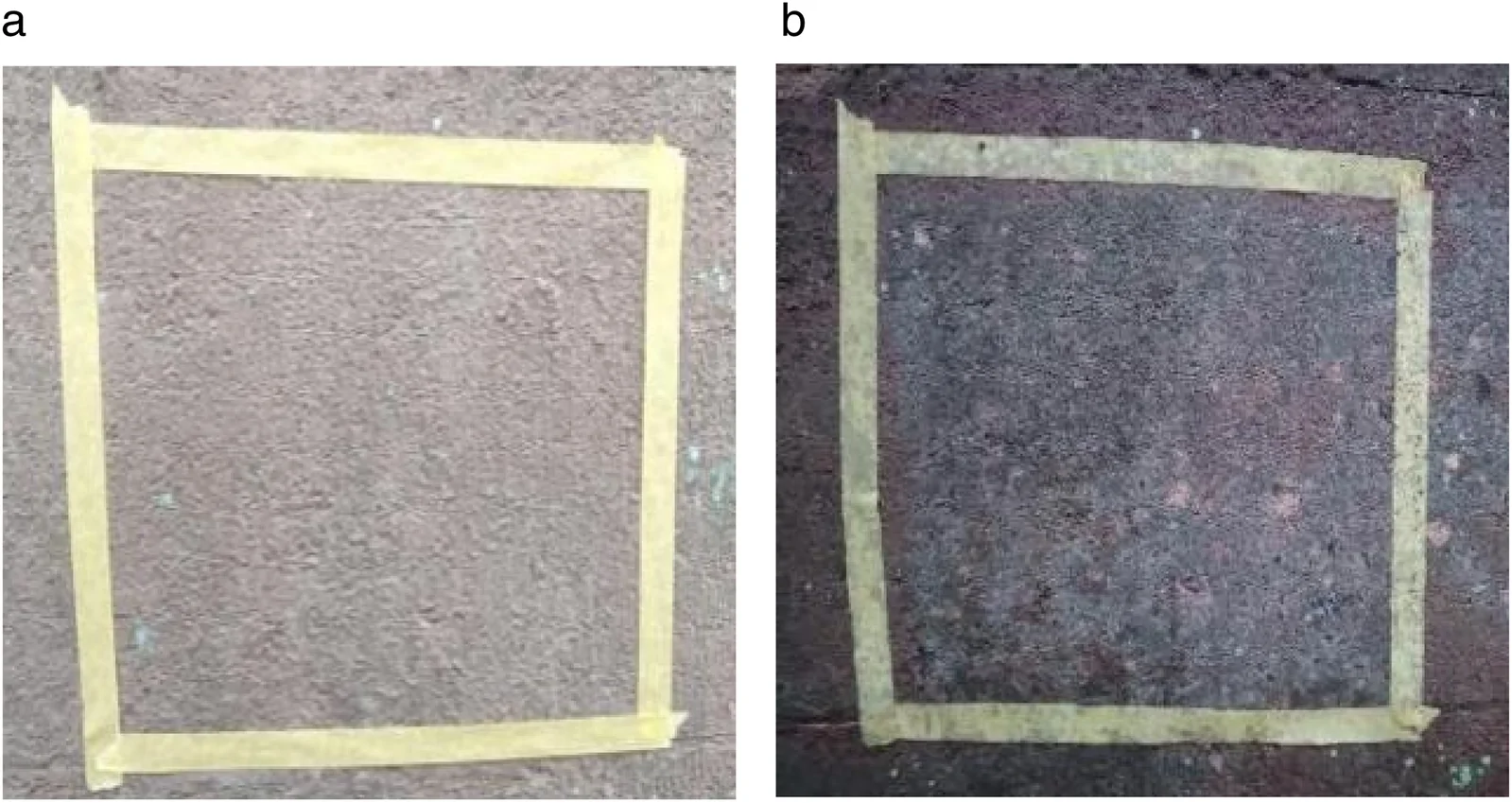
Cleaning results of crust on the surface of the Tomb of Yue Yun. (a) Tomb of Yue Yun-Before Cleaning, (b) Tomb of Yue Yun-After Cleaning.

#### 3.1.3. Results of cleaning surface water erosion.

The surfaces of the stone cultural relics in the tomb of Yue Fei area suffer from relatively severe water-induced contamination. Appropriate cleaning parameters should be selected according to the degree of pollution. Fig 5 illustrates the current state of water damage on four stone cultural relics at the tomb of Yue Fei. The Half-Moon Pond and the deck of the Loyalty Bridge show evident signs of water erosion, with the tested areas exhibiting heavy rust crusts. This is because these two sites are close to water sources, and rainwater erosion cannot easily dissipate. In contrast, the railings of the Loyalty Bridge and the surface of Yue Fei’s tombstone experience lighter water damage. This is due to the shelter provided by overlying plants or trees, which prevents most rainwater from directly falling on the rock surfaces. Additionally, the vertical orientation of the tombstone further reduces the impact of rainwater erosion on its lower sections.

**Fig 5:**
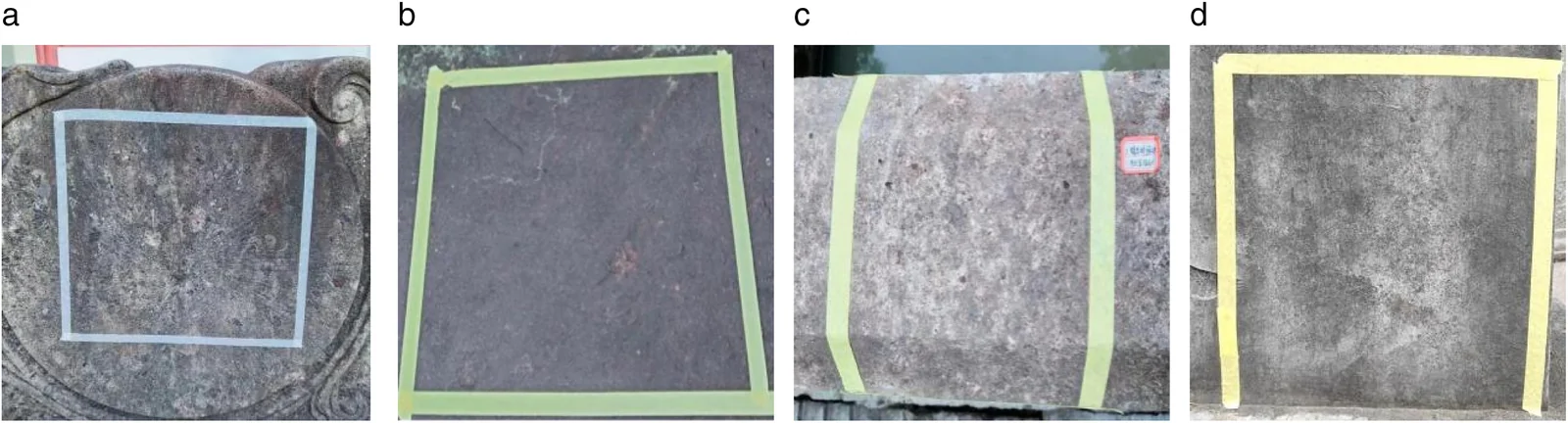
Current status of water erosion and contamination at the Tomb of Yue Fei. (a) Drum-Shaped stone at Half-Moon Pond, (b) Deck of Jingzhong Bridge, (c) Fence of Jingzhong Bridge, (d) Tombstone of Yue Yun.

Appropriate cleaning technical parameters were selected based on the degree of water erosion in four areas. For the Drum-Shaped stones at the Half-Moon Pond and the deck of the Jingzhong Bridge, the cleaning time was set between 3 and 5 minutes, with a cleaning distance of 1 cm and a cleaning angle of 30 degrees. For the fence of the Jingzhong Bridge and the Tombstone of Yue Yun, the cleaning time was set between 0.5 and 1 minute, with a cleaning distance of 3 cm and a cleaning angle of 90 degrees perpendicular to the surface. The cleaning pressure was uniformly maintained at 4 bar. The cleaning results are shown in Fig 6.

**Fig 6:**
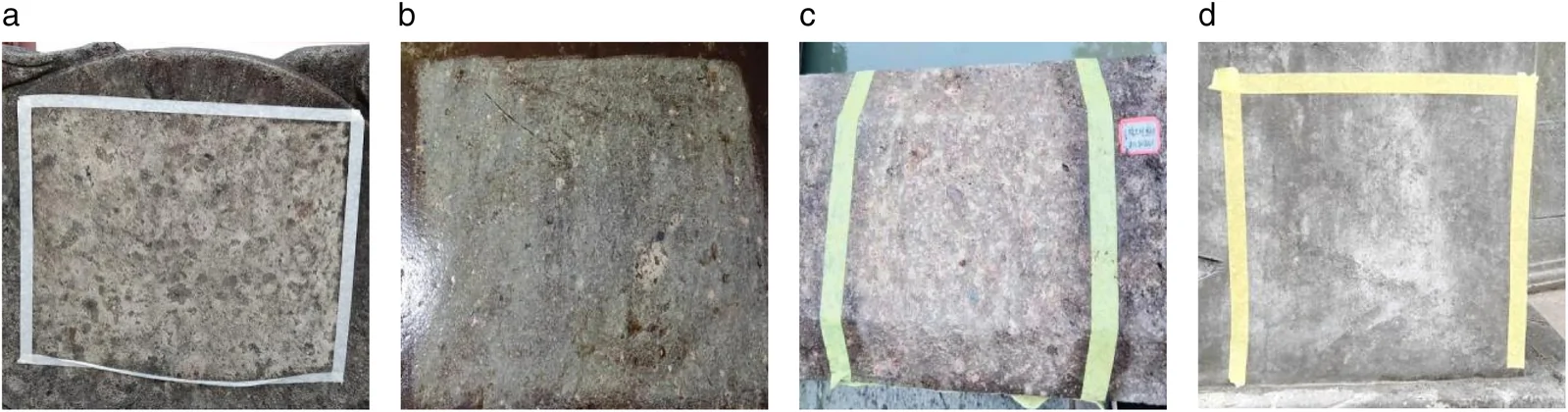
Post-cleaning image of water damage erosion at the Tomb of Yue Fei. (a) Drum-Shaped stone at Half-Moon Pond, (b) Deck of Jingzhong Bridge, (c) Fence of Jingzhong Bridge, (d) Tombstone of Yue Yun.

#### 3.1.4. Results of cleaning for microbial erosion.

Microbial erosion represents the second most extensive category of surface contamination affecting the stone cultural relics at the tomb of Yue Fei. Due to severe water damage in the surrounding area and dense vegetation, the degree of microbial erosion is considerable. The primary types of microbial contamination are moss, lichen, and mold. The current state of contamination is shown in Fig 7.

**Fig 7:**
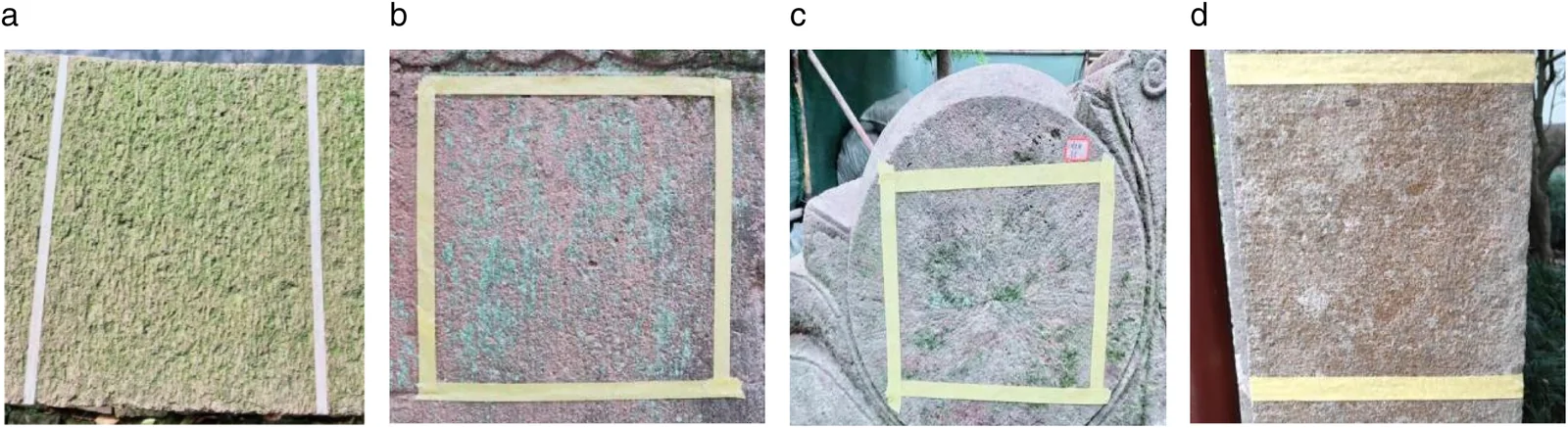
Current state of microbial erosion at the Tomb of Yue Fei. (a) Fence of Half-Moon Pond, (b) Tomb of Yue Yun, (c) Drum-Shaped stone at Jingzhong Bridge, (d) Side of Loyal and Filial Gate.

Appropriate technical parameters for cleaning can be selected based on the degree of different microbial contamination and its combination with other types of pollution. For instance, the rock surface at the fence of the Half-Moon Pond is rough; selecting a cleaning distance of 1 cm, a duration of 1 minute, an angle of 60°, and a pressure of 2 ~ 4 bar can effectively remove contaminants. The surface of Yue Yun’s tomb is covered with moss and soil crust; a cleaning angle of 15° ~ 30° is chosen, with the other parameters remaining the same as before. The rock surface at the drum-shaped stones at Jingzhong Bridge is rough and severely damaged by water; a cleaning duration of about 3 minutes is selected, with the other parameters unchanged. The mold on the surface at the side of Loyal and Filial Gate is of an adhesive type, and the pollutant structure is loose; selecting a cleaning angle of 90°and a duration of 0.5 minutes allows for rapid removal.Microscopic images taken after cleaning demonstrated that most fungal hyphae and moss root structures were eliminated. However, in areas with rough surfaces such as the crescent pool enclosure, a small amount of residual microorganisms remained in the surface depressions, indicating that a second cleaning procedure with a slightly increased angle may be required to achieve complete eradication.The results after microbial cleaning of the tomb of Yue Fei are shown in Fig 8.

**Fig 8:**
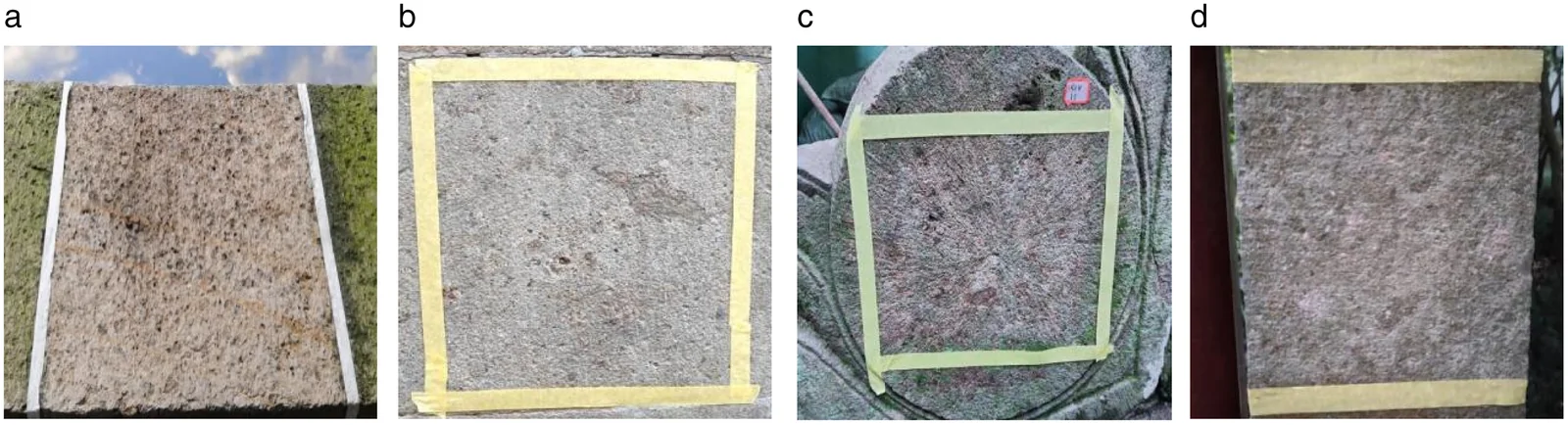
Post-Cleaning image of the Tomb of Yue Fei after microbial erosion treatment. (a) Fence of Half-Moon Pond, (b) Tomb of Yue Yun, (c) Drum-Shaped stone at Jingzhong Bridge, (d) Side of Loyal and Filial Gate.

#### 3.1.5. Cleaning results of artificial graffiti.

Among the stone cultural relics in the Tomb of Yue Fei area, artificial graffiti contamination is currently found only at one location: ink stains on the side of the Stele at the Jingzhong Cypress Pavilion. The stele has undergone multiple repairs, resulting in residual cement on its surface. The removal of the ink stains primarily relies on hot steam to activate the carbon particles and dissolve them in water. Therefore, the selected cleaning parameters were as follows: a horizontal cleaning angle of 90°, a distance of 3 ~ 5 cm, a cleaning duration of 0.5 minutes, and a cleaning pressure of 4 bar, which proved effective for removal. The results are shown in Fig 9.

**Fig 9:**
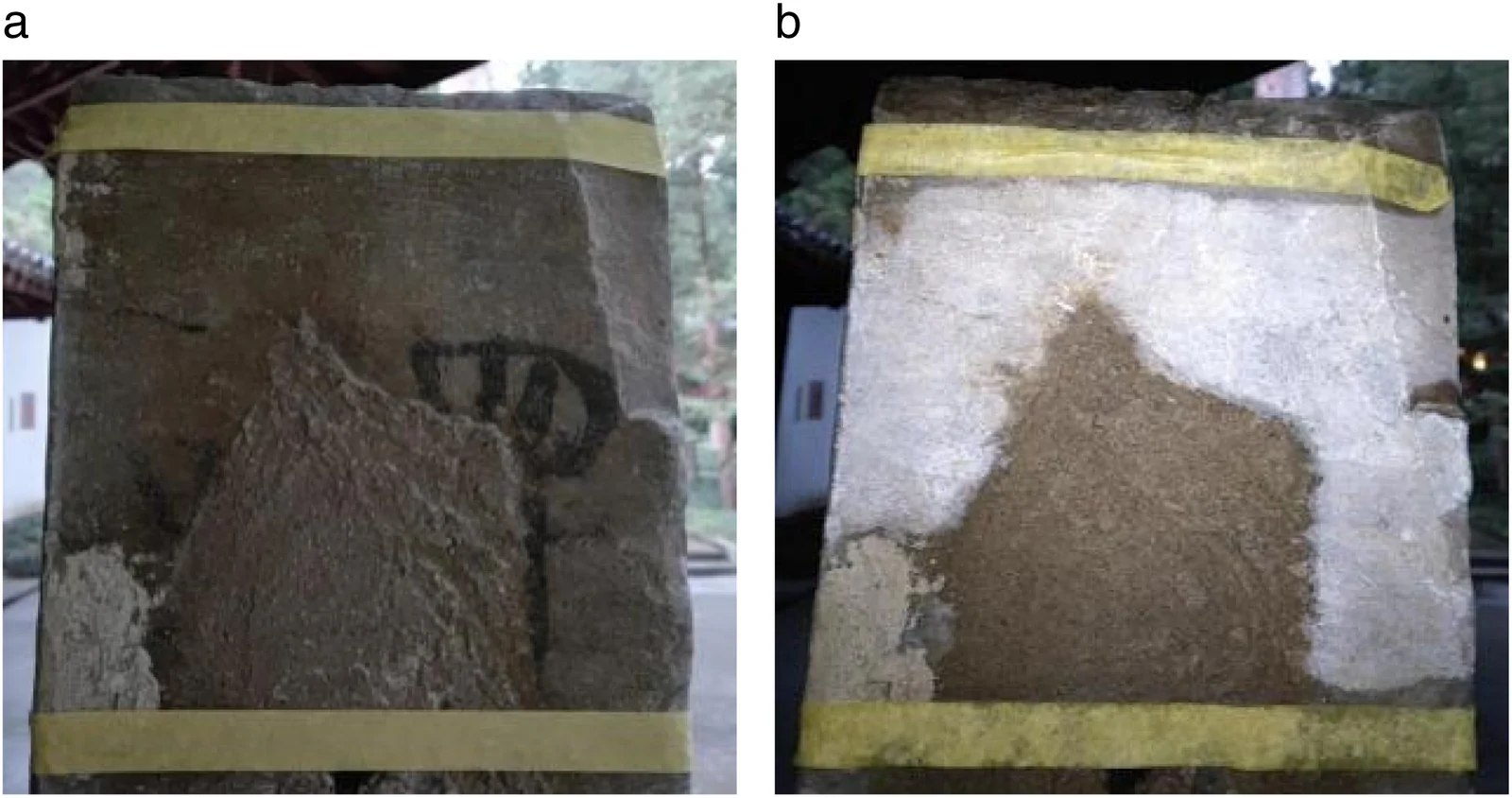
Cleaning results of the ink inscriptions on the stele at the Jingzhong Cypress Pavilion, tomb of Yue Fei. (a) Stele at the Jingzhong Cypress Pavilion-Before Cleaning, (b) Stele at the Jingzhong Cypress Pavilion-Afer Cleaning.

### 3.2. Analysis of the effect of cleaning test

#### 3.2.1. Infrared thermography inspection results.

Infrared thermography was employed to examine the temperature distribution on rock surfaces before and after steam cleaning, in order to analyze the potential damage caused by the cleaning process and assess the condition of the rock surface post‑cleaning. As shown in Fig 12, two warm zones appeared on the surface of the Yueyun tombstone after cleaning. This is attributed to hollowing phenomena within those areas and the fact that the cleaned section is located at the base of the tombstone.

The infrared detection results from Figs 10–14, comparing the rock surfaces before and after cleaning, indicate that the temperature distribution was relatively uniform prior to cleaning, with overall temperatures fluctuating around 30°C (within a range of 10°C). However, certain areas, such as the Drum-Shaped stone at Half-Moon Pond, exhibited higher temperatures (49°C). Two factors may explain this: first, the stone in that location was directly exposed to sunlight without any shading; second, the internal pore structure of the rock is relatively large, and some zones (shown as red areas) exhibit hollowing phenomena.

**Fig 10:**
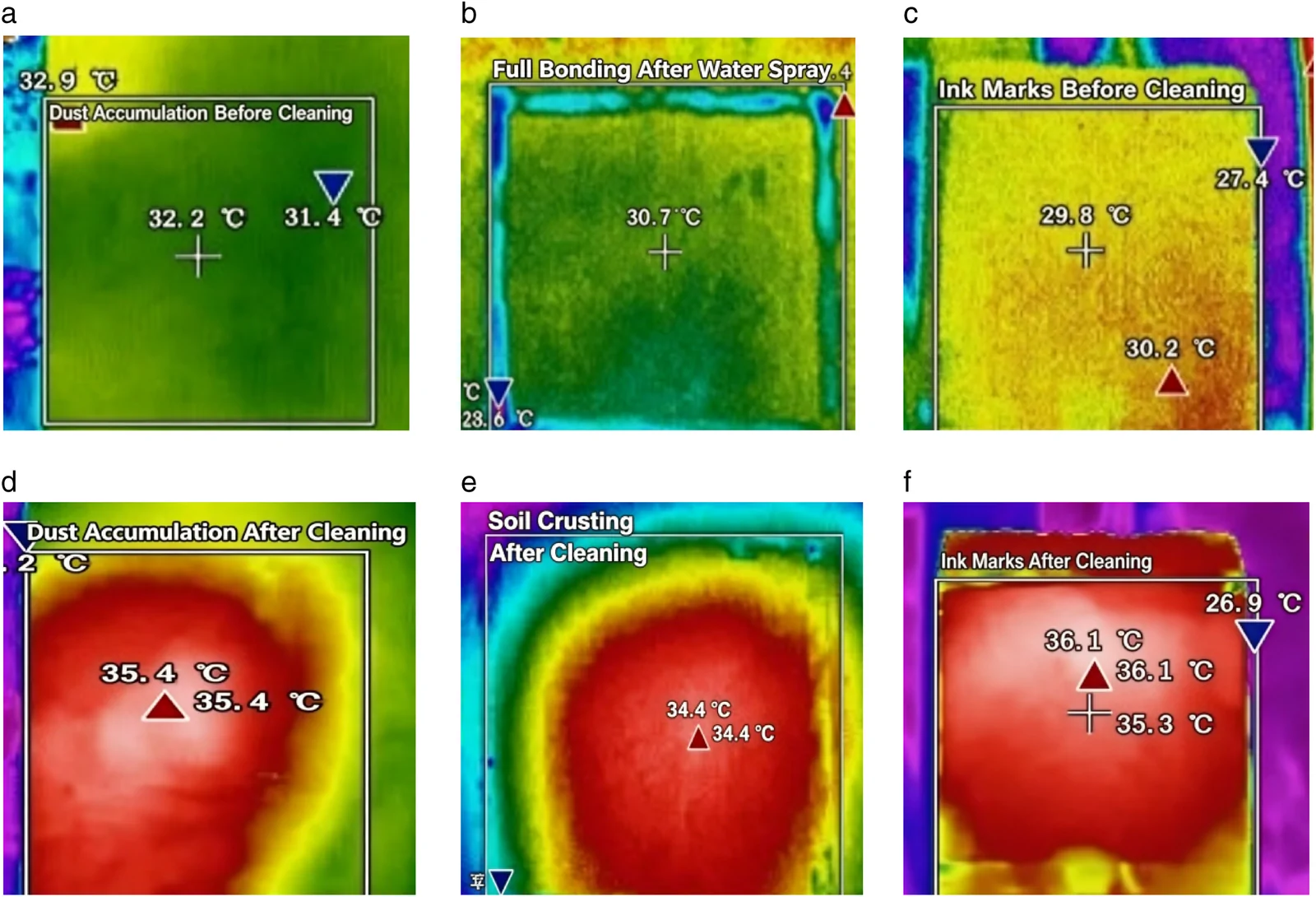
Infrared detection images of the surface contaminants on the stone cultural relics at the Tomb of Yue Fei before and after steam cleaning. (a) Before dust accumulation cleaning, (b) Before surface crust cleaning, (c) Before ink graffiti cleaning, (d) After dust accumulation cleaning, (e) After surface crust cleaning, (f) After ink graffiti cleaning.

**Fig 11:**
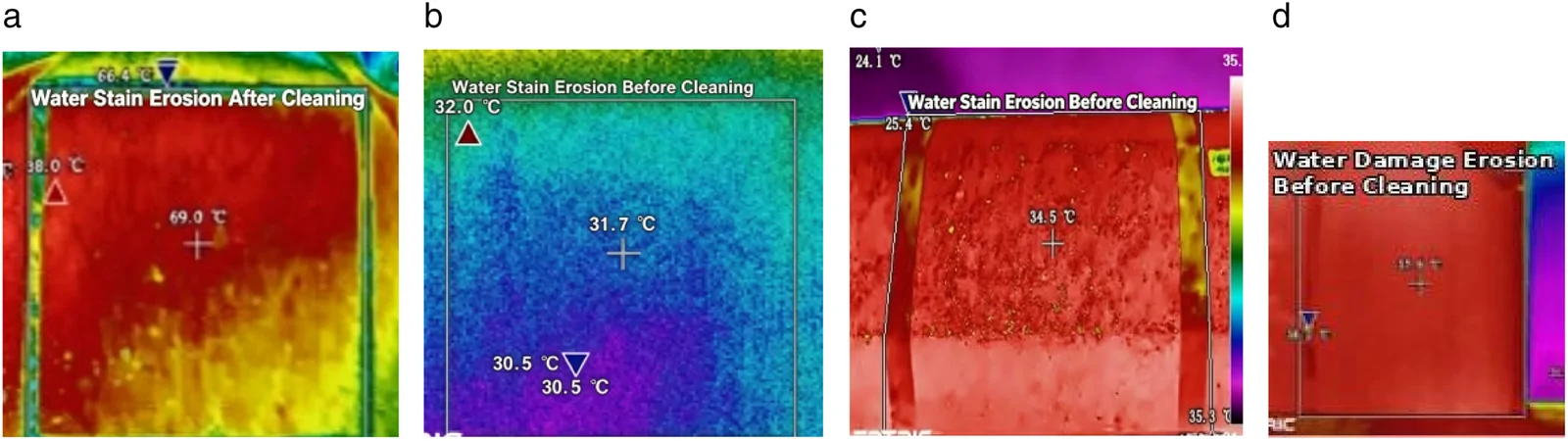
Infrared image of the Tomb of Yue Fei before cleaning of water damage erosion and contamination. (a) Drum-Shaped stone at Half-Moon Pond, (b) Deck of Jingzhong Bridge, (c) Fence of Jingzhong Bridge, (d) Tombstone of Yue Yun.

**Fig 12:**
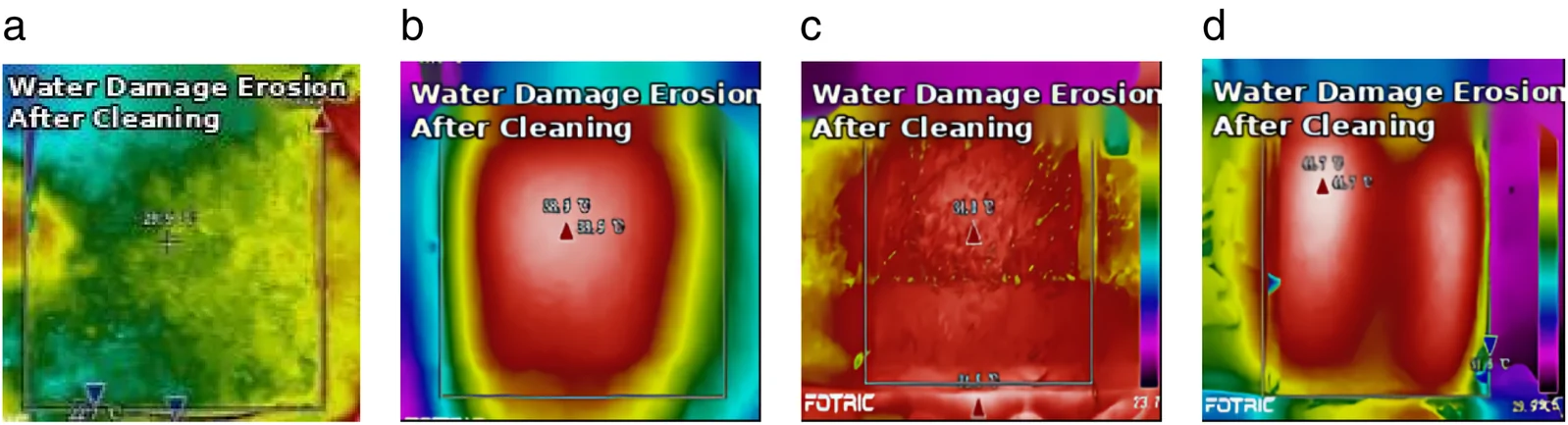
Infrared image of the Tomb of Yue Fei after cleaning of water damage erosion and contamination. (a) Drum-Shaped stone at Half-Moon Pond, (b) Deck of Jingzhong Bridge, (c) Fence of Jingzhong Bridge, (d) Tombstone of Yue Yun.

**Fig 13:**
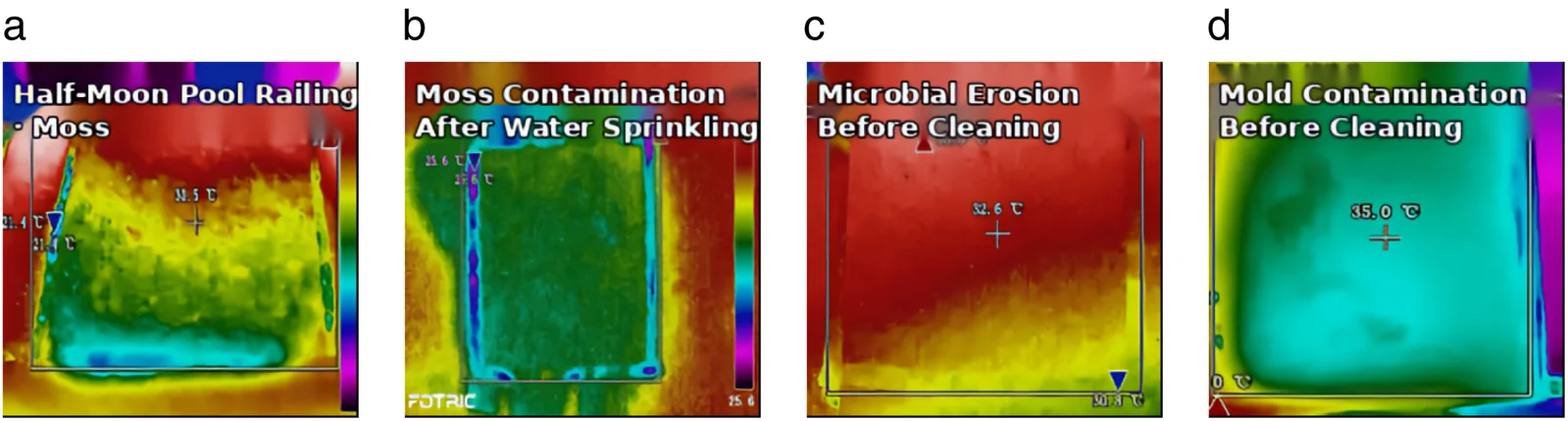
Infrared image of microbial erosion and Contamination before cleaning at the Tomb of Yue Fei. (a) Fence of Half-Moon Pond, (b) Tomb of Yue Yun, (c) Drum-Shaped stone at Jingzhong Bridge, (d) Side of Loyal and Filial Gate.

**Fig 14:**
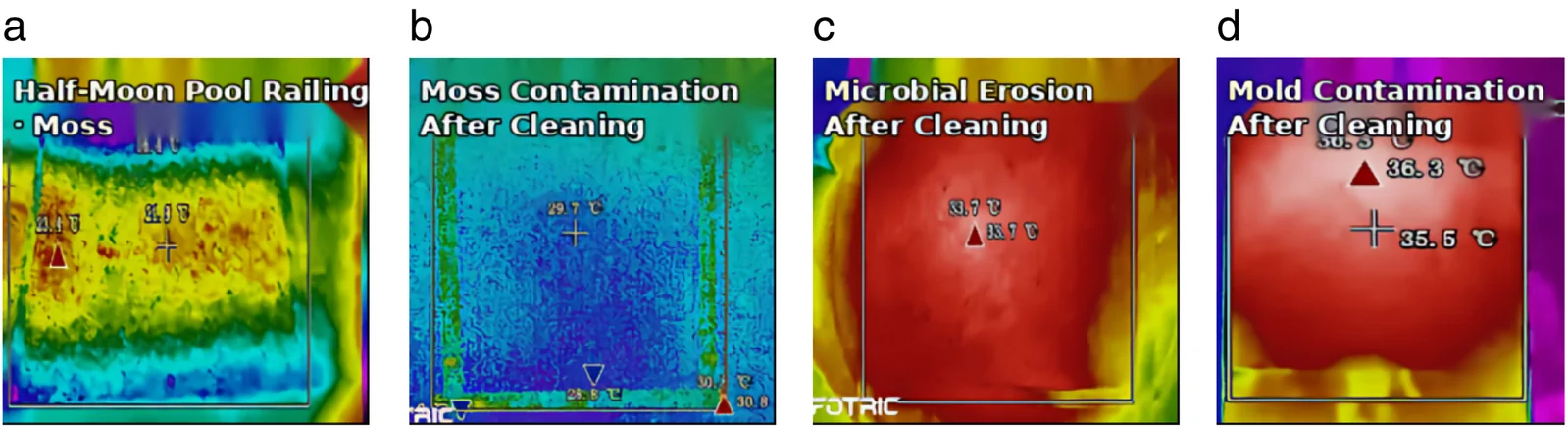
Infrared image of microbial erosion and contamination after cleaning at the Tomb of Yue Fei. (a) Fence of Half-Moon Pond, (b) Tomb of Yue Yun, (c) Drum-Shaped stone at Jingzhong Bridge, (d) Side of Loyal and Filial Gate.

#### 3.2.2. Calculation results of rock surface temperature change.

Infrared detection images were imported into the supporting AnalyzIR software to calculate the temperature change amplitude at the central point of the cleaned area before and after cleaning, with an interval of more than 30 minutes. The calculation results of temperature changes on the surface of stone cultural relics at the tomb of Yue Fei before and after steam cleaning of surface contaminants are presented in Table 3.

**Table 3:** Calculation results of temperature changes before and after cleaning of surface contaminants on stone cultural relics in the tomb of Yue Fei (unit: °C).

Types and regions of contaminants	Temperature before cleaning	Temperature after cleaning	Temperature difference	Temperature Range (%)
Dust accumulation-tombstone of Yue Fei	32.2	35.4	3.2	9.94
Surface encrustation-tomb of Yue Yun	30.7	34.4	3.7	12.05
Ink graffiti-Stele at the Jingzhong Cypress Pavilion	29.5	36.1	6.6	22.37
Hydro erosion-drum-shaped stone at Half-Moon Pond	39.9	49.0	9.1	18.57
Hydro erosion-deck of Jingzhong Bridge	31.2	33.9	2.7	8.65
Hydro erosion-fence of Jingzhong Bridge	34.0	34.5	0.5	1.45
Hydro erosion-tombstone of Yue Yun	37.9	44.7	6.8	17.94
Microbial erosion-fence of Half-Moon Pond	21.9	30.5	8.6	28.20
Microbial erosion-tomb of Yue Yun	27.58	29.7	2.12	7.69
Microbial erosion-drum-shaped stone at Jingzhong Bridge	32.6	33.7	1.1	3.37
Microbial erosion-side of Loyal and Filial Gate	35.0	35.5	0.5	1.43

As indicated by the detection and calculation results of rock surface temperature changes in 11 areas with five types of contaminants before and after cleaning in Table 3, the test block surfaces were covered by contaminants before cleaning, which hindered heat dissipation from the rocks. After cleaning, rock permeability was enhanced, leading to a decrease in the surface temperature of the test blocks. Furthermore, the smaller the temperature variation amplitude after cleaning, the better the cleaning effect and the higher the rock safety. Overall, the temperature differences and variation amplitudes in the 11 cleaned areas at the tomb of Yue Fei were relatively low, all within the safe tolerance range of the rock itself.

## 4. Discussion of steam cleaning test results

The bonding layers between surface contaminants and the rock substrate of stone cultural relics are structured as “contaminant surface layer - bonding layer between contaminant and rock matrix - rock matrix layer.” During steam cleaning, the contaminant surface layer is first wetted, followed by impact cleaning that reaches the rock matrix layer. Contaminant removal relies on the dissolution of contaminants by water and steam, as well as the scouring and friction exerted by the steam flow.

Selecting cleaning conditions based on the characteristics of different contaminants can enhance effectiveness. For instance, lower cleaning parameters are suitable for accumulated dust. For crusted water stain contamination, an appropriate cleaning angle should be chosen to utilize steam flow friction for removal. For anthropogenic ink graffiti, parameters should be selected according to the chemical composition. Marker stains that are difficult to dissolve in water require longer cleaning times and a low cleaning angle. For grease stains, parameters are chosen based on the formation period and the composition type of the surface contamination, with a cleaning time exceeding 3 minutes.

## 5. Conclusion

The significance of this study for the conservation of the Yue Fei Tomb lies in the following.It provides a scientifically validated,parameter-optimized cleaning protocol that minimizes risk to the original stone while effectively removing harmful contaminants.It introduces an infrared thermography-based evaluation method that allows on-site, non-invasive monitoring of cleaning outcomes, which is crucial for a site of national importance.The integration of colorimetry, microscopy, and thermography establishes a holistic assessment framework that can be adapted for other stone cultural relics,thereby contributing to the broader field of heritage conservation science.The successful application at the Yue Fei Tomb demonstrates that steam cleaning,combined with advanced diagnostic tools,can play a vital role in preserving the aesthetic and structural integrity of irreplaceable cultural heritage.

(1) Based on the removal efficacy and safety of steam cleaning technology for common contaminants,a recommended parameter table for steam cleaning typical pollutants on the surface of stone cultural relics is proposed, refer to Table 4.

**Table 4:** Recommended steam cleaning parameters for typical contaminants on limestone cultural relics surfaces.

Types of contaminants	Combination type	Recommended cleaning conditions				Assessment of cleaning results
		Cleaning pressure (bar)	Cleaning time (min)	Cleaning distance (cm)	Cleaning angle (°)	
Dust accumulation	Adsorption type	2 ~ 4	0.5 ~ 1	3 ~ 5	60 ~ 90	The cleaning effect is excellent, with no surface damage to the rock
Surface encrustation	Surface intrusion	2 ~ 4	1 ~ 3	1 ~ 3	30 ~ 60	The cleaning effect is good, with no damage to the rock
Ink graffiti	Intrusive type	2 ~ 4	0.5 ~ 1	0.5 ~ 1	60 ~ 90	The contaminants are completely removed, with no damage to the rock.
Marker pen graffiti	Tight coupling	4 ~ 6	>3	0.5 ~ 1	30 ~ 60	A significant amount of contaminant residue remains, with minor damage to the rock.
Oil contamination	Intrusive type	The effectiveness of contaminant removal is related to the contamination cycle, with longer cycles resulting in poorer removal performance. The rock exhibits minor damage. It is recommended to perform multiple cleaning cycles, with the duration of each cycle controlled to 1 minute. This approach can effectively remove oil contamination while also avoiding damage to the rock surface.				
smoke contamination	Tight coupling +Intrusive type	2 ~ 4	>3	1 ~ 3	30 ~ 90	A significant amount of contaminant residue remains, with minor damage to the rock.
Hydro erosion	Intrusive type	2 ~ 4	1 ~ 5	1 ~ 3	60 ~ 90	The cleaning effect is good, with no damage to the rock
Microbial contamination	Surface intrusion	4 ~ 6	1 ~ 3	1 ~ 3	60 ~ 90	The cleaning effect is excellent, with no surface damage to the rock
The cleaning target is limestone with a low degree of weathering, in which the calcite content exceeds 50%.

(2) A non-destructive evaluation method based on infrared thermography was established and validated.The temperature variation index T showed a strong correlation with color difference ΔE1 (R^2^ = 0.69) and a moderate correlation with surface roughness Sku (R^2^ = 0.46). After cleaning, temperature differences in 11 test areas ranged from 0.5 °C to 9.1 °C,with corresponding T values between 1.45% and 28.20%.All values remained within safe limits,confirming that the cleaning process did not cause thermal stress damage to the stone substrate.
(3) The integration of infrared thermography with complementary techniques—such as colorimetry and microscopic imaging—enables a multi-dimensional assessment of cleaning quality. This approach not only enhances the reliability of on-site evaluations but also provides a scientific basis for developing standardized cleaning protocols for stone cultural relics.
