# Supplementary material for: Infrared thermographic evaluation of cleaning stone relics at tomb of Yue Fei
Source: PLoS One. 2026 Oct 8;21(10):e0359835. doi: 10.1371/journal.pone.0359835 (PMC13648988; doi:10.1371/journal.pone.0359835)
Supplement: Supplementary file 4 [file pone.0359835.s004.docx]

# Response to Reviewers

Dear Editor and Reviewers,

We sincerely thank all reviewers for their careful reading and constructive comments on our manuscript. We have addressed each comment thoroughly and made corresponding revisions. The following is our point-by-point response to all reviewer comments. All revisions in the revised manuscript have been highlighted in red.

## Response to Reviewer #1

We greatly appreciate Reviewer #1's positive assessment and recognition of our systematic revisions. We have carefully addressed all the minor issues raised.

**Comment (1):** There are some different errors in the clean version and the modified version of the manuscript. For example, some references in the text are superscripts, and some are normal unmarked. Please check again to ensure that the contents of the two versions are unified. Similarly, the formula numbers 3-1 and 3-2 of the two versions do not match the chapter number 2.1.2. It is suggested that the full text should be unified chart and formula number, and the formula number should be (1), (2) ..., to remove the traces of the dissertation format.

**Response:** We have carefully reviewed both versions of the manuscript and made the following corrections:All formula numbers have been revised to use the format (1), (2), (3) consistently throughout the text, eliminating the previous chapter-based numbering system. The formula originally numbered as "3-1" and "3-2" in the previous version is now correctly numbered as formula (2) and (3) in Section 2.1.2. Formula (1) in Section 2.1.1 has also been verified and confirmed.

**Comment (2):** There are still a few irregularities in language expression. For example: Page 15 "The high coverage value Z of 55.3 %" does not define the meaning of "Z" and should be explained when it first appears (e.g. coverage value).

**Response:** We have added a clear definition of the coverage value Z when it first appears in Section 3.1.1. The revised sentence now reads:"The coverage value Z, defined as the percentage of the cleaned area exhibiting chromatic values within 5% of the reference clean stone surface, was 55.3%, indicating that most of the dust had been removed, restoring the original stone color."

**Comment (3):** There is a mixture of Chinese and English connectors in the text. There are many Chinese connectors "—" to connect sentences. Please check the full text to ensure the integrity of the sentence. In addition, the text size on Formula 3-2 seems to be the same as the size of the figure name, please check whether there is a mistake and modify it.

**Response:** We have thoroughly checked the entire manuscript and made the following corrections:All Chinese em-dashes "—" have been replaced with appropriate English punctuation (commas, semicolons, or periods) to ensure proper sentence structure and readability.The font size of formula (2) and (3) (previously 3-2) has been corrected to match the standard formula font size, distinct from figure caption formatting.We have also reviewed the entire text for any remaining language irregularities and made additional improvements where necessary.

## Response to Reviewer #4

We appreciate Reviewer #4's critical assessment, which has helped us further strengthen the manuscript. We address each point below:

**Comment (1):** The use of infrared thermography is not described well and the results are not convincing. I suggest to re-test the samples using the solar loading thermography approach introduced recently and already tested in the cultural heritage field. Also, raw thermal images should be processed with advanced algorithms in order to improve the quality of the results and, therefore, the message that the authors want to convey to the readers. As they are, the thermal images added to the text are not so significant; what about emissivity variation?

**Response:** We are deeply grateful to the reviewer for this expert and constructive suggestion. Solar loading thermography (SLT) is indeed a sophisticated and well-established technique, and we have studied the relevant literature with great admiration for its proven value in cultural heritage assessment. After thorough deliberation and careful evaluation of our site conditions, we respectfully note that, **due to several practical constraints specific to our study, the direct application of SLT would be extremely challenging**. We sincerely hope the reviewer will understand our position, which we explain below.

**(1) Objective limitations of the illumination conditions**

The fundamental strength of SLT lies in its reliance on prolonged, uniform solar exposure to establish stable thermal gradients over diurnal cycles. However, the Tomb of Yue Fei is a semi-open garden-style historic site, shaded by ancient cypress trees and architectural overhangs. The stone surfaces are consequently covered by **dappled and intermittent shadows** throughout the day, making consistent solar heating difficult to achieve. We are concerned that, under such conditions, thermal noise caused by irregular shading could mask genuine structural signals, potentially compromising the reliability of SLT data rather than enhancing it.

**(2) Differences in research focus**

SLT excels at detecting deep subsurface anomalies, such as delaminations and voids, as beautifully demonstrated in the studies cited by the reviewer. The primary objective of our study, however, is to **evaluate the immediate effects of localized, short-duration steam cleaning on surface patches** (typically 10–30 cm in size). Our active approach—applying controlled thermal stimulation through steam and monitoring recovery before and after cleaning—allows rapid acquisition of comparable data precisely at the cleaned locations. The two methods each have their own strengths, suited to different scales and diagnostic goals.

**(3) Operational constraints at an open heritage site**

As a nationally protected heritage site, the Tomb of Yue Fei remains open to the public, requiring us to balance conservation work with visitor access. SLT generally requires continuous monitoring over several hours or even days, which would be difficult to accommodate without significantly disrupting site operations and visitor circulation. During our field campaign, we were only able to work within small, cordoned areas during low-traffic periods, **making the extended, uninterrupted observation window required for SLT impractical**.

**(4) Cross-validation of our current approach**

We are encouraged to find that our thermographic results show good agreement with chromatic aberration measurements (R² = 0.69) and microscopic observations. This consistency suggests that our current method can reliably reflect cleaning efficacy within the scope of this study. Naturally, should future opportunities arise at more suitable sites, we would be very interested in employing SLT for deeper structural assessment.

(5) Regarding emissivity variation, we have now added the following clarification to Section 2.1.1:"To ensure the reliability of thermographic measurements, the emissivity of the stone surface was set to 0.95 based on literature values for limestone materials.To minimize the impact of solar radiation, all measurements were conducted in overcast or shaded areas, with environmental temperature and humidity recorded. The interval between thermal imaging before and after cleaning was maintained at approximately 30 minutes to ensure comparability of environmental conditions.

**Comment (2):** Many papers cited in the References section refer to chinese colleagues although important schools developed in the course of times about the research field here treated goes from France and Italy; I suggest to the authors to deepen the scientific literature.

**Response:** We agree with the reviewer that international contributions, particularly from French and Italian research groups, are essential to this field. In response, we have Expanded the literature review to include more works from French and Italian research schools.Enhanced the Introduction (Section 1) with a more comprehensive review of international contributions, now including specific references to French, Italian, and Greek research groups.

**Comment (3):** Thermographic results in sub-section 3.2.1 contain words written in chinese although they should be written in english.

**Response:** We apologize for this oversight. All Chinese text in the thermal images has been translated to English. The figure captions and descriptions in Section 3.2.1 now use consistent English terminology throughout.
