# Supplementary material for: Infrared thermographic evaluation of cleaning stone relics at tomb of Yue Fei
Source: PLoS One. 2026 Oct 8;21(10):e0359835. doi: 10.1371/journal.pone.0359835 (PMC13648988; doi:10.1371/journal.pone.0359835)
Supplement: Supplementary file 5 [file pone.0359835.s001.docx]

This study presents an innovative approach by employing steam cleaning combined with infrared thermographic inspection in the context of cultural relic conservation. It plays a significant role in guiding future cleaning procedures for the conservation and restoration of Yue Fei’s Tomb. However, from a research perspective, the current paper requires substantial revisions and should not be treated merely as a material selection process akin to that in a survey and design report. Specific revisions are outlined below:

1. The current title is overly broad and should be refined. A more scientifically precise and focused title, such as "Infrared Thermographic Evaluation of Cleaning Stone Relics at Yue Fei’ Tomb," would better highlight the study's contributions.

2. The color difference tests and microscopic observations mentioned in the abstract and main text lack corresponding evidence or validation in the body of the paper. These should be substantiated. Additionally, the purpose and significance of the content in Table 2 need to be clearly stated.

3. The introductory section should be structured under the heading "1. Introduction."

4. In the introductory chapter, the methodology for identifying the types of deterioration should be elaborated. The author should incorporate qualitative analytical techniques—such as XRD, XRF, FTIR, and macroscopic imaging—referenced in Table 1 to support the descriptions of deterioration.

5. The infrared thermography methodology section lacks details regarding the timing and light intensity before and after testing. It is well known that solar radiation affects infrared thermographic measurements of outdoor stone relics, leading to varying results at different times of day (e.g., sunrise, noon, sunset). The paper should clearly specify the testing constraints and ensure that the time intervals before and after cleaning are short and explicitly indicated.

6. While the efficacy of steam cleaning is widely recognized, the distinctive contribution of this study lies in its application of infrared thermography to assess the conservation of Yue Fei’s Tomb—a valuable historical and cultural heritage site. Therefore, the conclusions should be expanded in scope, with emphasis placed on key findings and novel insights. Furthermore, the significance of the testing methods in providing valuable experience for the conservation and restoration of Yue Fei’s Tomb should be explicitly discussed.
