# Supplementary material for: Infrared thermographic evaluation of cleaning stone relics at tomb of Yue Fei
Source: PLoS One. 2026 Oct 8;21(10):e0359835. doi: 10.1371/journal.pone.0359835 (PMC13648988; doi:10.1371/journal.pone.0359835)
Supplement: Supplementary file 6 [file pone.0359835.s002.docx]

1. The title was revised to "Infrared Thermographic Evaluation of Cleaning Stone Relics at Yue Fei's Tomb".

2. In the results section, specific chromaticity values or microscopic descriptions were added for each type of contamination to verify the findings. The purpose and significance were elaborated under the equipment table in section 2.2.

3. A new introduction chapter has been added.

4. In the introduction, the basis and method of identifying the deterioration type are described by combining qualitative analysis techniques.

5. Add a description of the infrared detection conditions in the Materials and Methods section.

6. The conclusion section has been reorganized into three main components. The first component presents recommended steam cleaning parameters for typical pollutants on stone artifacts. The second component establishes and validates a non-destructive testing method based on infrared thermal imaging. The third component demonstrates that the integration of infrared thermal imaging with complementary techniques such as colorimetry and microscopic imaging enables multidimensional evaluation of stone artifact cleaning quality.

7. The modified parts are marked in red in the revised draft.

8.Figure 2 is referenced in the text.

9.The field experiments were conducted by staff members of the **Administration of Yue Fei’Temple Scenic Area**, the official authority responsible for the management and conservation of the site. As the work was performed as part of routine conservation activities under the administration’s purview, no additional permits were required. All cleaning procedures were carried out in compliance with national cultural heritage protection regulations and under the supervision of the site management.
