# Supplementary material for: Infrared thermographic evaluation of cleaning stone relics at tomb of Yue Fei
Source: PLoS One. 2026 Oct 8;21(10):e0359835. doi: 10.1371/journal.pone.0359835 (PMC13648988; doi:10.1371/journal.pone.0359835)
Supplement: Peer review history

# Peer review: Decision Letter 0

**DOI:** 10.1371/journal.pone.0359835.r001

**Authors:** Benedetto Schiavo

17 Feb 2026

Dear Dr. Ye,

Thank you for submitting your manuscript to PLOS ONE. After careful consideration, we feel that it has merit but does not fully meet PLOS ONE’s publication criteria as it currently stands. Therefore, we invite you to submit a revised version of the manuscript that addresses the points raised during the review process.

The manuscript was evaluated by two reviewers who recognize the potential relevance of the study; however, several issues need to be addressed before the paper can be considered for publication. Please revise the manuscript by clarifying the novelty of the proposed approach through comparison with existing urine stabilization and nutrient recovery methods, explaining why conventional strategies are insufficient for HEU treatment. The discussion should be expanded to better explain the observed decline in urease activity and to propose possible operational or control strategies. A brief discussion on potential environmental risks related to reuse of the nutrient-rich effluent, including salinity accumulation and possible long-term ecological impacts, should also be included.

Please submit your revised manuscript by Apr 03 2026 11:59PM. If you will need more time than this to complete your revisions, please reply to this message or contact the journal office at plosone@plos.org. When you're ready to submit your revision, log on to https://www.editorialmanager.com/pone/ and select the 'Submissions Needing Revision' folder to locate your manuscript file.

- A letter that responds to each point raised by the academic editor and reviewer(s). You should upload this letter as a separate file labeled 'Response to Reviewers'.
- A marked-up copy of your manuscript that highlights changes made to the original version. You should upload this as a separate file labeled 'Revised Manuscript with Track Changes'.
- An unmarked version of your revised paper without tracked changes. You should upload this as a separate file labeled 'Manuscript'.

If you would like to make changes to your financial disclosure, please include your updated statement in your cover letter. Guidelines for resubmitting your figure files are available below the reviewer comments at the end of this letter.

If applicable, we recommend that you deposit your laboratory protocols in protocols.io to enhance the reproducibility of your results. Protocols.io assigns your protocol its own identifier (DOI) so that it can be cited independently in the future. For instructions see: https://journals.plos.org/plosone/s/submission-guidelines#loc-laboratory-protocols. Additionally, PLOS ONE offers an option for publishing peer-reviewed Lab Protocol articles, which describe protocols hosted on protocols.io. Read more information on sharing protocols at https://plos.org/protocols?utm_medium=editorial-email&utm_source=authorletters&utm_campaign=protocols.

We look forward to receiving your revised manuscript.

Kind regards,

Benedetto Schiavo, Ph.D.

Academic Editor

PLOS One

Journal Requirements:

When submitting your revision, we need you to address these additional requirements.

1. Please ensure that your manuscript meets PLOS ONE's style requirements, including those for file naming. The PLOS ONE style templates can be found at

https://journals.plos.org/plosone/s/file?id=wjVg/PLOSOne_formatting_sample_main_body.pdf and

https://journals.plos.org/plosone/s/file?id=ba62/PLOSOne_formatting_sample_title_authors_affiliations.pdf

2. Please note that PLOS One has specific guidelines on code sharing for submissions in which author-generated code underpins the findings in the manuscript. In these cases, we expect all author-generated code to be made available without restrictions upon publication of the work. Please review our guidelines at https://journals.plos.org/plosone/s/materials-and-software-sharing#loc-sharing-code and ensure that your code is shared in a way that follows best practice and facilitates reproducibility and reuse.

3. In your Methods section, please provide additional information regarding the permits you obtained for the work. Please ensure you have included the full name of the authority that approved the field site access and, if no permits were required, a brief statement explaining why.

4. Please update your submission to use the PLOS LaTeX template. The template and more information on our requirements for LaTeX submissions can be found at http://journals.plos.org/plosone/s/latex.

5. We note that your Data Availability Statement is currently as follows: “All relevant data are within the manuscript and its Supporting Information files.”

Please confirm at this time whether or not your submission contains all raw data required to replicate the results of your study. Authors must share the “minimal data set” for their submission. PLOS defines the minimal data set to consist of the data required to replicate all study findings reported in the article, as well as related metadata and methods (https://journals.plos.org/plosone/s/data-availability#loc-minimal-data-set-definition).

For example, authors should submit the following data:

- The values behind the means, standard deviations and other measures reported;

- The values used to build graphs;

- The points extracted from images for analysis.

Authors do not need to submit their entire data set if only a portion of the data was used in the reported study.

If your submission does not contain these data, please either upload them as Supporting Information files or deposit them to a stable, public repository and provide us with the relevant URLs, DOIs, or accession numbers. For a list of recommended repositories, please see https://journals.plos.org/plosone/s/recommended-repositories.

If there are ethical or legal restrictions on sharing a de-identified data set, please explain them in detail (e.g., data contain potentially sensitive information, data are owned by a third-party organization, etc.) and who has imposed them (e.g., an ethics committee). Please also provide contact information for a data access committee, ethics committee, or other institutional body to which data requests may be sent. If data are owned by a third party, please indicate how others may request data access.

6. PLOS requires an ORCID iD for the corresponding author in Editorial Manager on papers submitted after December 6th, 2016. Please ensure that you have an ORCID iD and that it is validated in Editorial Manager. To do this, go to ‘Update my Information’ (in the upper left-hand corner of the main menu), and click on the Fetch/Validate link next to the ORCID field. This will take you to the ORCID site and allow you to create a new iD or authenticate a pre-existing iD in Editorial Manager.

7. Please ensure that you refer to Figure 2 in your text as, if accepted, production will need this reference to link the reader to the figure.

8. If the reviewer comments include a recommendation to cite specific previously published works, please review and evaluate these publications to determine whether they are relevant and should be cited. There is no requirement to cite these works unless the editor has indicated otherwise.

[Note: HTML markup is below. Please do not edit.]

Reviewers' comments:

Reviewer's Responses to Questions

**Comments to the Author**

1. Is the manuscript technically sound, and do the data support the conclusions?

Reviewer #1: Yes

Reviewer #2: Partly

**********

2. Has the statistical analysis been performed appropriately and rigorously?

Reviewer #1: Yes

Reviewer #2: No

**********

3. Have the authors made all data underlying the findings in their manuscript fully available?

The PLOS Data policy

Reviewer #1: Yes

Reviewer #2: Yes

**********

4. Is the manuscript presented in an intelligible fashion and written in standard English?

Reviewer #1: Yes

Reviewer #2: Yes

**********

Reviewer #1: The manuscript presents a systematic and well-designed study on the integration of urease-producing bacteria (UPB)-mediated enzymatic hydrolysis with biological nitrification for nutrient recovery from low-pH hormone-extracted urine (HEU), a challenging and underexplored urine-derived pharmaceutical wastewater. Overall, this paper demonstrates certain value in both scientific and engineering terms.

1.Although the manuscript emphasizes the novelty of treating low-pH HEU, a more explicit comparison with existing urine stabilization and nutrient recovery approaches would strengthen the conceptual positioning. The author is advised to briefly explain in the introduction why conventional urine treatment strategies are insufficient for HEU.

2.The sharp decline in urease activity after day 18 is an important observation with direct engineering implications. However, the explanation remains largely speculative, focusing on microbial competition and metabolic imbalance.The authors could discuss potential process control strategies.

3.While the nutrient-rich effluent shows promise as a fertilizer, the manuscript does not sufficiently address potential environmental risks, such as: 1)Salinity accumulation in soils; 2)Long-term ecological impacts.The authors could add a brief discussion acknowledging these risks and outlining future work needed to evaluate environmental safety.

4.Ensure consistent use of terms such as “HEU,” “low-pH urine-derived wastewater,” and “hormone-extracted urine” throughout the manuscript.

5.Some complex schematic figures could benefit from simplified legends or higher resolution to improve readability.

6.Standardize units (e.g., mg N·L⁻¹·h⁻¹) and formatting throughout text, tables, and figures.

7.The manuscript is generally well written, but minor grammatical and stylistic refinements would further improve clarity, particularly in the Discussion section.

Reviewer #2: This study presents an innovative approach by employing steam cleaning combined with infrared thermographic inspection in the context of cultural relic conservation. It plays a significant role in guiding future cleaning procedures for the conservation and restoration of Yue Fei’s Tomb. However, from a research perspective, the current paper requires substantial revisions and should not be treated merely as a material selection process akin to that in a survey and design report. Specific revisions are outlined below:

1. The current title is overly broad and should be refined. A more scientifically precise and focused title, such as "Infrared Thermographic Evaluation of Cleaning Stone Relics at Yue Fei’ Tomb," would better highlight the study's contributions.

2. The color difference tests and microscopic observations mentioned in the abstract and main text lack corresponding evidence or validation in the body of the paper. These should be substantiated. Additionally, the purpose and significance of the content in Table 2 need to be clearly stated.

3. The introductory section should be structured under the heading "1. Introduction."

4. In the introductory chapter, the methodology for identifying the types of deterioration should be elaborated. The author should incorporate qualitative analytical techniques—such as XRD, XRF, FTIR, and macroscopic imaging—referenced in Table 1 to support the descriptions of deterioration.

5. The infrared thermography methodology section lacks details regarding the timing and light intensity before and after testing. It is well known that solar radiation affects infrared thermographic measurements of outdoor stone relics, leading to varying results at different times of day (e.g., sunrise, noon, sunset). The paper should clearly specify the testing constraints and ensure that the time intervals before and after cleaning are short and explicitly indicated.

6. While the efficacy of steam cleaning is widely recognized, the distinctive contribution of this study lies in its application of infrared thermography to assess the conservation of Yue Fei’s Tomb—a valuable historical and cultural heritage site. Therefore, the conclusions should be expanded in scope, with emphasis placed on key findings and novel insights. Furthermore, the significance of the testing methods in providing valuable experience for the conservation and restoration of Yue Fei’s Tomb should be explicitly discussed.

**********

what does this mean?). If published, this will include your full peer review and any attached files.

If you choose “no”, your identity will remain anonymous but your review may still be made public.

**Do you want your identity to be public for this peer review?** For information about this choice, including consent withdrawal, please see our Privacy Policy

Reviewer #1: No

Reviewer #2: No

**********

[NOTE: If reviewer comments were submitted as an attachment file, they will be attached to this email and accessible via the submission site. Please log into your account, locate the manuscript record, and check for the action link "View Attachments". If this link does not appear, there are no attachment files.]

To ensure your figures meet our technical requirements, please review our figure guidelines: https://journals.plos.org/plosone/s/figures

You may also use PLOS’s free figure tool, NAAS, to help you prepare publication quality figures: https://journals.plos.org/plosone/s/figures#loc-tools-for-figure-preparation.

NAAS will assess whether your figures meet our technical requirements by comparing each figure against our figure specifications.

Attachment Submitted filename: Review attachment.docx

# Author comment: Author response to Decision Letter 1

**DOI:** 10.1371/journal.pone.0359835.r002

3 Jun 2026

1. The title was revised to "Infrared Thermographic Evaluation of Cleaning Stone Relics at Yue Fei's Tomb".

2. In the results section, specific chromaticity values or microscopic descriptions were added for each type of contamination to verify the findings. The purpose and significance were elaborated under the equipment table in section 2.2.

3. A new introduction chapter has been added.

4. In the introduction, the basis and method of identifying the deterioration type are described by combining qualitative analysis techniques.

5. Add a description of the infrared detection conditions in the Materials and Methods section.

6. The conclusion section has been reorganized into three main components. The first component presents recommended steam cleaning parameters for typical pollutants on stone artifacts. The second component establishes and validates a non-destructive testing method based on infrared thermal imaging. The third component demonstrates that the integration of infrared thermal imaging with complementary techniques such as colorimetry and microscopic imaging enables multidimensional evaluation of stone artifact cleaning quality.

7. The modified parts are marked in red in the revised draft.

Attachment Submitted filename: response to revivewers.docx

# Peer review: Decision Letter 1

**DOI:** 10.1371/journal.pone.0359835.r003

**Authors:** Benedetto Schiavo

29 Jul 2026

Dear Dr. Ye,

Thank you for submitting your manuscript to PLOS ONE. After careful consideration, we feel that it has merit but does not fully meet PLOS ONE’s publication criteria as it currently stands. Therefore, we invite you to submit a revised version of the manuscript that addresses the points raised during the review process.

The reviewer considers that the infrared thermography methodology is insufficiently described and that the current thermographic results are not sufficiently convincing. The reviewer recommends re-evaluating the samples using the recently developed solar loading thermography approach, processing the raw thermal images with advanced image-processing algorithms, and discussing the potential influence of emissivity variations on the results. The reviewer also suggests including temporal plots showing the evolution of temperature and relative humidity together with the improved thermographic results. Furthermore, the literature review should be expanded to include relevant contributions from well-established research groups, particularly from France and Italy, in order to provide a more balanced overview of the field. Finally, all labels and annotations appearing in Chinese in the thermographic images of Section 3.2.1 should be translated into English.

Please submit your revised manuscript by Sep 12 2026 11:59PM. If you will need more time than this to complete your revisions, please reply to this message or contact the journal office at plosone@plos.org. When you're ready to submit your revision, log on to https://www.editorialmanager.com/pone/ and select the 'Submissions Needing Revision' folder to locate your manuscript file.

- A letter that responds to each point raised by the academic editor and reviewer(s). You should upload this letter as a separate file labeled 'Response to Reviewers'.
- A marked-up copy of your manuscript that highlights changes made to the original version. You should upload this as a separate file labeled 'Revised Manuscript with Track Changes'.
- An unmarked version of your revised paper without tracked changes. You should upload this as a separate file labeled 'Manuscript'.

If you would like to make changes to your financial disclosure, please include your updated statement in your cover letter. Guidelines for resubmitting your figure files are available below the reviewer comments at the end of this letter.

If applicable, we recommend that you deposit your laboratory protocols in protocols.io to enhance the reproducibility of your results. Protocols.io assigns your protocol its own identifier (DOI) so that it can be cited independently in the future. For instructions see: https://journals.plos.org/plosone/s/submission-guidelines#loc-laboratory-protocols. Additionally, PLOS ONE offers an option for publishing peer-reviewed Lab Protocol articles, which describe protocols hosted on protocols.io. Read more information on sharing protocols at https://plos.org/protocols?utm_medium=editorial-email&utm_source=authorletters&utm_campaign=protocols.

As the corresponding author, your ORCID iD is verified in the submission system and will appear in the published article. PLOS supports the use of ORCID, and we encourage all coauthors to register for an ORCID iD and use it as well. Please encourage your coauthors to verify their ORCID iD within the submission system before final acceptance, as unverified ORCID iDs will not appear in the published article. *Only* the individual author can complete the verification step; PLOS staff *cannot* verify ORCID iDs on behalf of authors.

We look forward to receiving your revised manuscript.

Kind regards,

Benedetto Schiavo, Ph.D.

Academic Editor

PLOS One

Journal Requirements:

If the reviewer comments include a recommendation to cite specific previously published works, please review and evaluate these publications to determine whether they are relevant and should be cited. There is no requirement to cite these works unless the editor has indicated otherwise.

[Note: HTML markup is below. Please do not edit.]

Reviewers' comments:

Reviewer's Responses to Questions

**Comments to the Author**

Reviewer #1: All comments have been addressed

Reviewer #2: (No Response)

Reviewer #3: All comments have been addressed

Reviewer #4: (No Response)

**********

2. Is the manuscript technically sound, and do the data support the conclusions?

Reviewer #1: Yes

Reviewer #2: No

Reviewer #3: Yes

Reviewer #4: No

**********

3. Has the statistical analysis been performed appropriately and rigorously?

Reviewer #1: Yes

Reviewer #2: Yes

Reviewer #3: Yes

Reviewer #4: No

**********

4. Have the authors made all data underlying the findings in their manuscript fully available?

The PLOS Data policy

Reviewer #1: Yes

Reviewer #2: Yes

Reviewer #3: Yes

Reviewer #4: No

**********

5. Is the manuscript presented in an intelligible fashion and written in standard English?

Reviewer #1: Yes

Reviewer #2: Yes

Reviewer #3: Yes

Reviewer #4: No

**********

Reviewer #1: The author has made serious and systematic revisions to the first-instance opinions, and the quality of the manuscript has been significantly improved. The main improvements include: increasing the analysis data of pollutant composition, clarifying the environmental control conditions of infrared detection, supplementing the quantitative indicators such as color difference in the result part, rewriting the conclusion to make it more hierarchical, and improving the equipment parameter description. At present, the paper has achieved high scientific standardization and readability, the experimental method is basically clear, and the supporting relationship between data analysis and conclusion is obviously strengthened. Therefore, it is recommended to be accepted after the author completes the following minor revisions.

(1) There are some different errors in the clean version and the modified version of the manuscript. For example, some references in the text are superscripts, and some are normal unmarked. Please check again to ensure that the contents of the two versions are unified. Similarly, the formula numbers 3-1 and 3-2 of the two versions do not match the chapter number 2.1.2. It is suggested that the full text should be unified chart and formula number, and the formula number should be (1), (2) ..., to remove the traces of the dissertation format.

(2) There are still a few irregularities in language expression. For example: Page 15 “The high coverage value Z of 55.3 %” does not define the meaning of “Z” and should be explained when it first appears (e.g. coverage value).

(3) There is a mixture of Chinese and English connectors in the text. There are many Chinese connectors “ — ”to connect sentences. Please check the full text to ensure the integrity of the sentence. In addition, the text size on Formula 3-2 seems to be the same as the size of the figure name, please check whether there is a mistake and modify it.

Reviewer #2: (No Response)

Reviewer #3: This manuscript has been thoroughly revised based on the previous comments. I have no further comments and recommend publication.

Reviewer #4: ^ The use of indrared thermography is not described well and the results are not convincing.

I suggest to re-test the samples using the solar loading thermography approach introduced recently and already tested in the cultural heritage field. Also, raw thermal images should be processed with advanced algorithms in order to improve the quality of the results and, therefore, the message that the authors want to convey to the readers.

As they are, the thermal images added to the text are not so significant; what about emissivity variation?

^ A trend in time (graphs) of the temperature and relative humidity variations should also be added along with the improved thermographic results acquired with the suggested method (solar loading thermography);

^ Many papers cited in the References section refer to chinese colleagues although important schools developed in the course of times about the research field here treated goes from France and Italy; I suggest to the authors to deepen the scientific literature;

^ Thermographic results in sub-section 3.2.1 contain words written in chinese although they should be written in english.

**********

what does this mean?). If published, this will include your full peer review and any attached files.

If you choose “no”, your identity will remain anonymous but your review may still be made public.

**Do you want your identity to be public for this peer review?** For information about this choice, including consent withdrawal, please see our Privacy Policy

Reviewer #1: No

Reviewer #2: No

Reviewer #3: No

Reviewer #4: No

**********

[NOTE: If reviewer comments were submitted as an attachment file, they will be attached to this email and accessible via the submission site. Please log into your account, locate the manuscript record, and check for the action link "View Attachments". If this link does not appear, there are no attachment files.]

To ensure your figures meet our technical requirements, please review our figure guidelines: https://journals.plos.org/plosone/s/figures

You may also use PLOS’s free figure tool, NAAS, to help you prepare publication quality figures: https://journals.plos.org/plosone/s/figures#loc-tools-for-figure-preparation.

NAAS will assess whether your figures meet our technical requirements by comparing each figure against our figure specifications.

# Author comment: Author response to Decision Letter 2

**DOI:** 10.1371/journal.pone.0359835.r004

2 Sep 2026

Response to Reviewer #1

We greatly appreciate Reviewer #1's positive assessment and recognition of our systematic revisions. We have carefully addressed all the minor issues raised.

Comment (1): There are some different errors in the clean version and the modified version of the manuscript. For example, some references in the text are superscripts, and some are normal unmarked. Please check again to ensure that the contents of the two versions are unified. Similarly, the formula numbers 3-1 and 3-2 of the two versions do not match the chapter number 2.1.2. It is suggested that the full text should be unified chart and formula number, and the formula number should be (1), (2) ..., to remove the traces of the dissertation format.

Response: We have carefully reviewed both versions of the manuscript and made the following corrections:All formula numbers have been revised to use the format (1), (2), (3) consistently throughout the text, eliminating the previous chapter-based numbering system. The formula originally numbered as "3-1" and "3-2" in the previous version is now correctly numbered as formula (2) and (3) in Section 2.1.2. Formula (1) in Section 2.1.1 has also been verified and confirmed.

Comment (2): There are still a few irregularities in language expression. For example: Page 15 "The high coverage value Z of 55.3 %" does not define the meaning of "Z" and should be explained when it first appears (e.g. coverage value).

Response: We have added a clear definition of the coverage value Z when it first appears in Section 3.1.1. The revised sentence now reads:"The coverage value Z, defined as the percentage of the cleaned area exhibiting chromatic values within 5% of the reference clean stone surface, was 55.3%, indicating that most of the dust had been removed, restoring the original stone color."

Comment (3): There is a mixture of Chinese and English connectors in the text. There are many Chinese connectors "—" to connect sentences. Please check the full text to ensure the integrity of the sentence. In addition, the text size on Formula 3-2 seems to be the same as the size of the figure name, please check whether there is a mistake and modify it.

Response: We have thoroughly checked the entire manuscript and made the following corrections:All Chinese em-dashes "—" have been replaced with appropriate English punctuation (commas, semicolons, or periods) to ensure proper sentence structure and readability.The font size of formula (2) and (3) (previously 3-2) has been corrected to match the standard formula font size, distinct from figure caption formatting.We have also reviewed the entire text for any remaining language irregularities and made additional improvements where necessary.

Response to Reviewer #4

We appreciate Reviewer #4's critical assessment, which has helped us further strengthen the manuscript. We address each point below:

Comment (1): The use of infrared thermography is not described well and the results are not convincing. I suggest to re-test the samples using the solar loading thermography approach introduced recently and already tested in the cultural heritage field. Also, raw thermal images should be processed with advanced algorithms in order to improve the quality of the results and, therefore, the message that the authors want to convey to the readers. As they are, the thermal images added to the text are not so significant; what about emissivity variation?

Response: We are deeply grateful to the reviewer for this expert and constructive suggestion. Solar loading thermography (SLT) is indeed a sophisticated and well-established technique, and we have studied the relevant literature with great admiration for its proven value in cultural heritage assessment. After thorough deliberation and careful evaluation of our site conditions, we respectfully note that, due to several practical constraints specific to our study, the direct application of SLT would be extremely challenging. We sincerely hope the reviewer will understand our position, which we explain below.

(1) Objective limitations of the illumination conditions

The fundamental strength of SLT lies in its reliance on prolonged, uniform solar exposure to establish stable thermal gradients over diurnal cycles. However, the Tomb of Yue Fei is a semi-open garden-style historic site, shaded by ancient cypress trees and architectural overhangs. The stone surfaces are consequently covered by dappled and intermittent shadows throughout the day, making consistent solar heating difficult to achieve. We are concerned that, under such conditions, thermal noise caused by irregular shading could mask genuine structural signals, potentially compromising the reliability of SLT data rather than enhancing it.

(2) Differences in research focus

SLT excels at detecting deep subsurface anomalies, such as delaminations and voids, as beautifully demonstrated in the studies cited by the reviewer. The primary objective of our study, however, is to evaluate the immediate effects of localized, short-duration steam cleaning on surface patches (typically 10–30 cm in size). Our active approach—applying controlled thermal stimulation through steam and monitoring recovery before and after cleaning—allows rapid acquisition of comparable data precisely at the cleaned locations. The two methods each have their own strengths, suited to different scales and diagnostic goals.

(3) Operational constraints at an open heritage site

As a nationally protected heritage site, the Tomb of Yue Fei remains open to the public, requiring us to balance conservation work with visitor access. SLT generally requires continuous monitoring over several hours or even days, which would be difficult to accommodate without significantly disrupting site operations and visitor circulation. During our field campaign, we were only able to work within small, cordoned areas during low-traffic periods, making the extended, uninterrupted observation window required for SLT impractical.

(4) Cross-validation of our current approach

We are encouraged to find that our thermographic results show good agreement with chromatic aberration measurements (R² = 0.69) and microscopic observations. This consistency suggests that our current method can reliably reflect cleaning efficacy within the scope of this study. Naturally, should future opportunities arise at more suitable sites, we would be very interested in employing SLT for deeper structural assessment.

(5)Regarding emissivity variation, we have now added the following clarification to Section 2.1.1:"To ensure the reliability of thermographic measurements, the emissivity of the stone surface was set to 0.95 based on literature values for limestone materials.To minimize the impact of solar radiation, all measurements were conducted in overcast or shaded areas, with environmental temperature and humidity recorded. The interval between thermal imaging before and after cleaning was maintained at approximately 30 minutes to ensure comparability of environmental conditions.

Comment (2): Many papers cited in the References section refer to chinese colleagues although important schools developed in the course of times about the research field here treated goes from France and Italy; I suggest to the authors to deepen the scientific literature.

Response: We agree with the reviewer that international contributions, particularly from French and Italian research groups, are essential to this field. In response, we have Expanded the literature review to include more works from French and Italian research schools.Enhanced the Introduction (Section 1) with a more comprehensive review of international contributions, now including specific references to French, Italian, and Greek research groups.

Comment (3): Thermographic results in sub-section 3.2.1 contain words written in chinese although they should be written in english.

Response: We apologize for this oversight. All Chinese text in the thermal images has been translated to English. The figure captions and descriptions in Section 3.2.1 now use consistent English terminology throughout.

Attachment Submitted filename: Response to Reviewers.docx

# Peer review: Decision Letter 2

**DOI:** 10.1371/journal.pone.0359835.r005

**Authors:** Benedetto Schiavo

7 Sep 2026

Dear Dr. Ye,

Thank you for submitting your manuscript to PLOS One. After careful consideration, we feel that it has merit but does not fully meet PLOS One’s publication criteria as it currently stands. Therefore, we invite you to submit a revised version of the manuscript that addresses the points raised during the review process.

Please submit your revised manuscript by Oct 22 2026 11:59PM. If you will need more time than this to complete your revisions, please reply to this message or contact the journal office at plosone@plos.org. When you're ready to submit your revision, log on to https://www.editorialmanager.com/pone/ and select the 'Submissions Needing Revision' folder to locate your manuscript file.

- A letter that responds to each point raised by the academic editor and reviewer(s). You should upload this letter as a separate file labeled 'Response to Reviewers'.
- A marked-up copy of your manuscript that highlights changes made to the original version. You should upload this as a separate file labeled 'Revised Manuscript with Track Changes'.
- An unmarked version of your revised paper without tracked changes. You should upload this as a separate file labeled 'Manuscript'.

If you would like to make changes to your financial disclosure, please include your updated statement in your cover letter. Guidelines for resubmitting your figure files are available below the reviewer comments at the end of this letter.

If applicable, we recommend that you deposit your laboratory protocols in protocols.io to enhance the reproducibility of your results. Protocols.io assigns your protocol its own identifier (DOI) so that it can be cited independently in the future. For instructions see: https://journals.plos.org/plosone/s/submission-guidelines#loc-laboratory-protocols. Additionally, PLOS One offers an option for publishing peer-reviewed Lab Protocol articles, which describe protocols hosted on protocols.io. Read more information on sharing protocols at https://plos.org/protocols?utm_medium=editorial-email&utm_source=authorletters&utm_campaign=protocols.

As the corresponding author, your ORCID iD is verified in the submission system and will appear in the published article. PLOS supports the use of ORCID, and we encourage all coauthors to register for an ORCID iD and use it as well. Please encourage your coauthors to verify their ORCID iD within the submission system before final acceptance, as unverified ORCID iDs will not appear in the published article. *Only* the individual author can complete the verification step; PLOS staff *cannot* verify ORCID iDs on behalf of authors.

We look forward to receiving your revised manuscript.

Kind regards,

Benedetto Schiavo, Ph.D.

Academic Editor

PLOS One

Journal Requirements:

If the reviewer comments include a recommendation to cite specific previously published works, please review and evaluate these publications to determine whether they are relevant and should be cited. There is no requirement to cite these works unless the editor has indicated otherwise.

Please review your reference list to ensure that it is complete and correct. If you have cited papers that have been retracted, please include the rationale for doing so in the manuscript text, or remove these references and replace them with relevant current references. Any changes to the reference list should be mentioned in the rebuttal letter that accompanies your revised manuscript. If you need to cite a retracted article, indicate the article’s retracted status in the References list and also include a citation and full reference for the retraction notice.

[Note: HTML markup is below. Please do not edit.]

Reviewers' comments:

Reviewer's Responses to Questions

**Comments to the Author**

Reviewer #4: (No Response)

**********

2. Is the manuscript technically sound, and do the data support the conclusions?

Reviewer #4: Yes

**********

3. Has the statistical analysis been performed appropriately and rigorously?

Reviewer #4: N/A

**********

4. Have the authors made all data underlying the findings in their manuscript fully available?

The PLOS Data policy

Reviewer #4: Yes

**********

5. Is the manuscript presented in an intelligible fashion and written in standard English?

Reviewer #4: No

**********

Reviewer #4: ^ English language should be improved;

^ Ref [36] is not needed; replace it with more recent papers published into the field of solar loading thermography & cultural heritage (check, e.g. the years 2024-2025-2026).

**********

what does this mean?). If published, this will include your full peer review and any attached files.

If you choose “no”, your identity will remain anonymous but your review may still be made public.

**Do you want your identity to be public for this peer review?** For information about this choice, including consent withdrawal, please see our Privacy Policy

Reviewer #4: No

**********

[NOTE: If reviewer comments were submitted as an attachment file, they will be attached to this email and accessible via the submission site. Please log into your account, locate the manuscript record, and check for the action link "View Attachments". If this link does not appear, there are no attachment files.]

To ensure your figures meet our technical requirements, please review our figure guidelines: https://journals.plos.org/plosone/s/figures

You may also use PLOS’s free figure tool, NAAS, to help you prepare publication quality figures: https://journals.plos.org/plosone/s/figures#loc-tools-for-figure-preparation.

NAAS will assess whether your figures meet our technical requirements by comparing each figure against our figure specifications.

# Author comment: Author response to Decision Letter 3

**DOI:** 10.1371/journal.pone.0359835.r006

12 Sep 2026

Response to Reviewers

Dear Editor and Reviewers,

We sincerely thank all reviewers for their careful reading and constructive comments on our manuscript. We have addressed each comment thoroughly and made corresponding revisions. The following is our point-by-point response to all reviewer comments. All revisions in the revised manuscript have been highlighted in red.

Response to Reviewer #1

We greatly appreciate Reviewer #1's positive assessment and recognition of our systematic revisions. We have carefully addressed all the minor issues raised.

Comment (1): There are some different errors in the clean version and the modified version of the manuscript. For example, some references in the text are superscripts, and some are normal unmarked. Please check again to ensure that the contents of the two versions are unified. Similarly, the formula numbers 3-1 and 3-2 of the two versions do not match the chapter number 2.1.2. It is suggested that the full text should be unified chart and formula number, and the formula number should be (1), (2) ..., to remove the traces of the dissertation format.

Response: We have carefully reviewed both versions of the manuscript and made the following corrections:All formula numbers have been revised to use the format (1), (2), (3) consistently throughout the text, eliminating the previous chapter-based numbering system. The formula originally numbered as "3-1" and "3-2" in the previous version is now correctly numbered as formula (2) and (3) in Section 2.1.2. Formula (1) in Section 2.1.1 has also been verified and confirmed.

Comment (2): There are still a few irregularities in language expression. For example: Page 15 "The high coverage value Z of 55.3 %" does not define the meaning of "Z" and should be explained when it first appears (e.g. coverage value).

Response: We have added a clear definition of the coverage value Z when it first appears in Section 3.1.1. The revised sentence now reads:"The coverage value Z, defined as the percentage of the cleaned area exhibiting chromatic values within 5% of the reference clean stone surface, was 55.3%, indicating that most of the dust had been removed, restoring the original stone color."

Comment (3): There is a mixture of Chinese and English connectors in the text. There are many Chinese connectors "—" to connect sentences. Please check the full text to ensure the integrity of the sentence. In addition, the text size on Formula 3-2 seems to be the same as the size of the figure name, please check whether there is a mistake and modify it.

Response: We have thoroughly checked the entire manuscript and made the following corrections:All Chinese em-dashes "—" have been replaced with appropriate English punctuation (commas, semicolons, or periods) to ensure proper sentence structure and readability.The font size of formula (2) and (3) (previously 3-2) has been corrected to match the standard formula font size, distinct from figure caption formatting.We have also reviewed the entire text for any remaining language irregularities and made additional improvements where necessary.

Response to Reviewer #4

We appreciate Reviewer #4's critical assessment, which has helped us further strengthen the manuscript. We address each point below:

Comment (1): The use of infrared thermography is not described well and the results are not convincing. I suggest to re-test the samples using the solar loading thermography approach introduced recently and already tested in the cultural heritage field. Also, raw thermal images should be processed with advanced algorithms in order to improve the quality of the results and, therefore, the message that the authors want to convey to the readers. As they are, the thermal images added to the text are not so significant; what about emissivity variation?

Response: We are deeply grateful to the reviewer for this expert and constructive suggestion. Solar loading thermography (SLT) is indeed a sophisticated and well-established technique, and we have studied the relevant literature with great admiration for its proven value in cultural heritage assessment. After thorough deliberation and careful evaluation of our site conditions, we respectfully note that, due to several practical constraints specific to our study, the direct application of SLT would be extremely challenging. We sincerely hope the reviewer will understand our position, which we explain below.

(1) Objective limitations of the illumination conditions

The fundamental strength of SLT lies in its reliance on prolonged, uniform solar exposure to establish stable thermal gradients over diurnal cycles. However, the Tomb of Yue Fei is a semi-open garden-style historic site, shaded by ancient cypress trees and architectural overhangs. The stone surfaces are consequently covered by dappled and intermittent shadows throughout the day, making consistent solar heating difficult to achieve. We are concerned that, under such conditions, thermal noise caused by irregular shading could mask genuine structural signals, potentially compromising the reliability of SLT data rather than enhancing it.

(2) Differences in research focus

SLT excels at detecting deep subsurface anomalies, such as delaminations and voids, as beautifully demonstrated in the studies cited by the reviewer. The primary objective of our study, however, is to evaluate the immediate effects of localized, short-duration steam cleaning on surface patches (typically 10–30 cm in size). Our active approach—applying controlled thermal stimulation through steam and monitoring recovery before and after cleaning—allows rapid acquisition of comparable data precisely at the cleaned locations. The two methods each have their own strengths, suited to different scales and diagnostic goals.

(3) Operational constraints at an open heritage site

As a nationally protected heritage site, the Tomb of Yue Fei remains open to the public, requiring us to balance conservation work with visitor access. SLT generally requires continuous monitoring over several hours or even days, which would be difficult to accommodate without significantly disrupting site operations and visitor circulation. During our field campaign, we were only able to work within small, cordoned areas during low-traffic periods, making the extended, uninterrupted observation window required for SLT impractical.

(4) Cross-validation of our current approach

We are encouraged to find that our thermographic results show good agreement with chromatic aberration measurements (R² = 0.69) and microscopic observations. This consistency suggests that our current method can reliably reflect cleaning efficacy within the scope of this study. Naturally, should future opportunities arise at more suitable sites, we would be very interested in employing SLT for deeper structural assessment.

(5)Regarding emissivity variation, we have now added the following clarification to Section 2.1.1:"To ensure the reliability of thermographic measurements, the emissivity of the stone surface was set to 0.95 based on literature values for limestone materials.To minimize the impact of solar radiation, all measurements were conducted in overcast or shaded areas, with environmental temperature and humidity recorded. The interval between thermal imaging before and after cleaning was maintained at approximately 30 minutes to ensure comparability of environmental conditions.

Comment (2): Many papers cited in the References section refer to chinese colleagues although important schools developed in the course of times about the research field here treated goes from France and Italy; I suggest to the authors to deepen the scientific literature.

Response: We agree with the reviewer that international contributions, particularly from French and Italian research groups, are essential to this field. In response, we have Expanded the literature review to include more works from French and Italian research schools.Enhanced the Introduction (Section 1) with a more comprehensive review of international contributions, now including specific references to French, Italian, and Greek research groups.

Comment (3): Thermographic results in sub-section 3.2.1 contain words written in chinese although they should be written in english.

Response: We apologize for this oversight. All Chinese text in the thermal images has been translated to English. The figure captions and descriptions in Section 3.2.1 now use consistent English terminology throughout.

Attachment Submitted filename: Response_to_Reviewers_auresp_3.docx

# Editor report: Decision Letter 3

**DOI:** 10.1371/journal.pone.0359835.r007

**Authors:** Benedetto Schiavo

20 Sep 2026

Infrared Thermographic Evaluation of Cleaning Stone Relics at Tomb of Yue Fei

PONE-D-26-01793R3

Dear Dr. Ye,

We’re pleased to inform you that your manuscript has been judged scientifically suitable for publication and will be formally accepted for publication once it meets all outstanding technical requirements.

Within one week, you’ll receive an e-mail detailing the required amendments. When these have been addressed, you’ll receive a formal acceptance letter and your manuscript will be scheduled for publication.

An invoice will be generated when your article is formally accepted. Please note, if your institution has a publishing partnership with PLOS and your article meets the relevant criteria, all or part of your publication costs will be covered. Please make sure your user information is up-to-date by logging into Editorial Manager at Editorial Manager® and clicking the ‘Update My Information' link at the top of the page. For questions related to billing, please contact billing support.

If your institution or institutions have a press office, please notify them about your upcoming paper to help maximize its impact. If they’ll be preparing press materials, please inform our press team as soon as possible -- no later than 48 hours after receiving the formal acceptance. Your manuscript will remain under strict press embargo until 2 pm Eastern Time on the date of publication. For more information, please contact onepress@plos.org.

Kind regards,

Benedetto Schiavo, Ph.D.

Academic Editor

PLOS One

Additional Editor Comments (optional):

Reviewers' comments:

# Editor report: Acceptance letter

**DOI:** 10.1371/journal.pone.0359835.r008

**Authors:** Benedetto Schiavo

PONE-D-26-01793R3

PLOS One

Dear Dr. Ye,

I'm pleased to inform you that your manuscript has been deemed suitable for publication in PLOS One. Congratulations! Your manuscript is now being handed over to our production team.

At this stage, our production department will prepare your paper for publication. This includes ensuring the following:

* All references, tables, and figures are properly cited

* All relevant supporting information is included in the manuscript submission,

* There are no issues that prevent the paper from being properly typeset

You will receive further instructions from the production team, including instructions on how to review your proof when it is ready. Please keep in mind that we are working through a large volume of accepted articles, so please give us a few days to review your paper and let you know the next and final steps.

Lastly, if your institution or institutions have a press office, please let them know about your upcoming paper now to help maximize its impact. If they'll be preparing press materials, please inform our press team within the next 48 hours. Your manuscript will remain under strict press embargo until 2 pm Eastern Time on the date of publication. For more information, please contact onepress@plos.org.

You will receive an invoice from PLOS for your publication fee after your manuscript has reached the completed accept phase. If you receive an email requesting payment before acceptance or for any other service, this may be a phishing scheme. Learn how to identify phishing emails and protect your accounts at https://explore.plos.org/phishing.

If we can help with anything else, please email us at customercare@plos.org.

Thank you for submitting your work to PLOS One and supporting open access.

Kind regards,

PLOS One Editorial Office Staff

on behalf of

Dr. Benedetto Schiavo

Academic Editor

PLOS One
